# Photochemical Degradation of Dimethylmercury in Natural Waters

**DOI:** 10.1021/acs.est.1c08443

**Published:** 2022-04-20

**Authors:** Johannes West, Sonja Gindorf, Sofi Jonsson

**Affiliations:** Department of Environmental Science, Stockholm University, 106 91 Stockholm, Sweden

**Keywords:** methylmercury, demethylation, Baltic, Arctic, UV, monomethylmercury, sunlight, demethylation

## Abstract

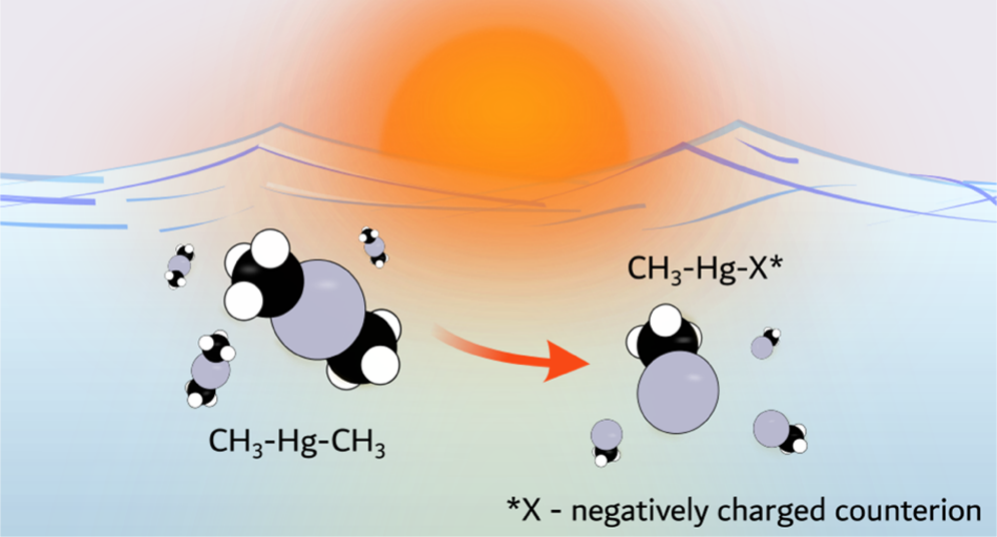

Photochemical demethylation of dimethylmercury (DMHg) could potentially be an important source of monomethylmercury (MMHg) in sunlit water. Whether or not DMHg is photochemically degraded when dissolved in water is, however, debated. While an early study suggested DMHg dissolved in natural waters to readily degrade, later work claimed DMHg to be stable in seawater under natural sunlight and that early observations may be due to experimental artifacts. Here, we present experimental data showing that DMHg is readily degraded by photochemical processes in different natural waters (including water from a DOC-rich stream, the Baltic Sea, and the Arctic Ocean) as well as in artificial seawater and purified water. For most of the waters, the degradation rate constant (*k*_d_) for DMHg measured in indoor experiments exceeded, or was close to, the *k*_d_ observed for MMHg. Outdoor incubations of DMHg in purified water and Arctic Ocean surface water further confirmed that DMHg is photochemically degraded under natural sunlight. Our study shows that DMHg is photochemically degraded in a range of natural waters and that this process may be a source of MMHg in sunlit waters where the supply or formation of DMHg is sufficient.

## Abstract 2

Degradation of dimethylmercury by sunlight may serve as an important source of bioaccumulating monomethylmercury in surface oceans.

## Introduction

Mercury (Hg) is a global pollutant released from the bedrock through natural and anthropogenic processes. While Hg is released into the environment as inorganic divalent Hg (Hg^II^ and elemental Hg (Hg^0^), the risks of Hg cycling in the environment are associated with the accumulation of monomethylmercury (CH_3_Hg-X, where X represents a counter anion, hereafter referred to as MMHg) in aquatic food webs. However, our understanding of the processes controlling the concentrations of MMHg in aquatic organisms, and thus the environmental risk of Hg, is incomplete. One of the important knowledge gaps to close is the unknown role of dimethylmercury ((CH_3_)_2_Hg, hereafter referred to as DMHg) in the marine biogeochemical cycle of Hg. Dimethylmercury is a volatile organomercurial found throughout marine environments. Although external sources are unlikely to explain the DMHg present, its formation pathways, as well as the stability of DMHg in marine waters, remain unknown. DMHg itself is not believed to bioaccumulate to concentrations of concern; however, DMHg could act as an important source of MMHg. Gaseous evasion of DMHg from marine surface waters and the subsequent photochemical demethylation of DMHg in the atmosphere has, for example, been suggested as a source of MMHg in the terrestrial system.^1,2^ Photodemethylation of dissolved DMHg could, in turn, represent a direct source of MMHg available for bioaccumulation in surface seawaters. Existing support for the latter comes from a study conducted by Mason and Sullivan, where surface waters with added DMHg were incubated onboard during a cruise in the South and Equatorial Atlantic.^3^ In their experiments, a greater loss of DMHg was observed in samples exposed to sunlight compared to those incubated in the dark. Additionally, a buildup of MMHg was observed in one of the water samples.^3^ These early incubations were done using Teflon bottles, which were later reported to be unsuitable for containing DMHg.^4^ Black et al. thus argued that the stability of DMHg in seawater remained unresolved and conducted sunlight exposure experiments using borosilicate glass flasks and DMHg containing seawater from Monterey Bay, California (no additions of DMHg were done). Based on the apparent stability of DMHg, even when exposed to sunlight, they argued DMHg in seawater to not readily be photodegraded under natural sunlight. They did, however, acknowledge that the discrepancy between the two studies also could be attributed to differences in water chemistry, biological activity, or differences in wavelengths transmitted through sample containers used. In addition, a post-hoc power analysis on the data by Black et al. showed that a DMHg photodemethylation rate as significant as evasion of DMHg from surface waters could not be excluded.^5^

To resolve whether or not DMHg is photodegradable, we synthesized an isotopically labeled DMHg tracer, which was then used to study the stability and potential degradation products of DMHg in contrasting waters (purified water, artificial seawater, and surface waters collected from the Arctic Ocean, the Baltic Sea, and a forest stream) when exposed to artificial as well as natural sunlight. The relevance of the demethylation rates gained was then evaluated by comparing demethylation rates of DMHg with the demethylation rates of an isotopically enriched MMHg tracer.

## Methods

### Sample Collection

The Arctic Ocean seawater was collected on the 11th of September onboard the icebreaker Oden during the SWEDARCTIC 2018 expedition. The sample was collected from a water depth of 5 m using conventional Niskin bottles mounted on a CTD rosette and was stored and transported frozen to the laboratory in Stockholm, Sweden. Baltic Sea surface water was sampled onboard R/V Electra in June 2021 from Landsort Deep (the deepest location in the Baltic Sea, 58.59822°N; 18.23304°E) at a water depth of 5 m using a 60 L go-flo bottle. The sample was filled into a 10 L canister and stored under dark conditions at ∼4 °C. The surface water collected was well oxygenated (∼7.9 mL L^–1^) and had a salinity of ∼6‰. The streamwater was collected manually from a stream in Täby, Stockholm (coordinates: 59°27.430′N, 18°4.558′E) and filtered through 0.45 μm syringe filters (Sarstedt Filtropur). The Baltic Sea surface water and streamwater samples were stored at 4 °C until used for the experiments (Supporting Information Table S1).

Artificial seawater was prepared by dissolving ca 35 g L^–1^ of aquarium salt (Instant Ocean) in Milli-Q water (>18.2 MΩ cm). The salinity was measured using a salinity meter (HCO 304, VWR) after the solutions had been stirred for 2 h using a magnetic stirrer. To remove excess salt particles, the artificial seawater was filtrated through 0.2 μm syringe filters (Sarstedt Filtropur). The salinity and pH in the two batches prepared were lower and higher than expected, respectively (salinity in batch 1: 30.7‰, salinity in batch 2: 29.2‰, expected: 35‰. pH in batch 2: 9.5 (Supporting Information Table S2)). This was derived to be caused by incomplete dissolution of the salt. For the experiments described below, the prepared artificial seawater is still assumed to largely have properties representative of natural seawaters.

### Reagents and Standards

DMHg is an extremely toxic compound that quickly permeates regular laboratory gloves and adsorbs through the skin. In 1997, a professor at Dartmouth College tragically passed away due to DMHg exposure after spilling a few drops of concentrated DMHg on her hand while wearing regular laboratory gloves (silver-shielded gloves have since this accident been recommended when working with DMHg).^6^ In our previous studies, working with highly concentrated DMHg has been avoided by synthesizing less concentrated DMHg solutions (typically in the range of a few ppm).^7,8^ The previous protocol used, however, results in residues of the photochemically active solvent tetrahydrofuran (THF) in the DMHg solutions. We, therefore, developed a new synthetization protocol to produce a DMHg standard free of potentially interfering compounds, detailed in the Supporting Information. MM^200^Hg and MM^201^Hg stock solutions were prepared from isotopically enriched ^200^Hg^II^ and ^201^Hg^II^ stock solutions (CortecNet). MMHg calibration solutions were prepared from MMHgCl stock solutions (1000 ppm MMHg standard, Alfa Aesar). All acids and bases used in the experiments were of trace metal grade. Acetate buffer and sodium tetraethyl borate (NaTEB) were prepared as described elsewhere.^8^

### Photochemical Degradation Experiments

Laboratory experiments were performed using a high-intensity UV lamp (Osram SUPRATEC HTC 400-241). The lamp was selected for its relatively good agreement with the relative intensity of sunlight in the UVA + UVB spectrum, but it differs notably by emitting light <290 nm, absent in sunlight at the Earth’s surface (Supporting Information Figure S1). The direct absorption of light by the molecules of DMHg and MMHg in the water phase at wavelengths not present in natural sunlight has been previously reported.^9^ To avoid introducing processes without environmental relevance, sample flasks were mounted in black boxes made out of plastic. The light was only let in through a hole in the front of the box, fitted with 2-inch Newport colored glass alternative (CGA) filters with a 50% cutoff at 305 nm. This way, wavelengths not present in natural sunlight were efficiently cut off. While the light intensity varied with the position of the lamp and between experiments, measurements revealed that samples were exposed to 15–20 W m^–2^ of light in the 305–390 nm range (LUTRON model UV 340A). In addition to experiments with 305 nm filters, 320 nm filters were used to evaluate the impact of the UVB region of light on Hg species photodecomposition (Supporting Information Figure S1). The lamp was cooled with a strong flow of pressurized air directed at the lamp. Sample flask temperature measurements revealed that the temperature was below 40 °C for time points where *k*_d_ was determined (within the first 150 min of the experiments).

All experiments were conducted with custom-made sample containers made from 2.5 mm clear quartz glass with about 90% light transmission throughout the natural light spectrum (ilmasil PN, Supporting Information Figure S1). For experiments a–l (Supporting Information Table S1), flat-sided cylinders were used, 55 mm (2 inches) in diameter and 60 cm in length, giving a total volume of ca 114 mL. The cylinders were acid-washed and rinsed well before each experiment. DM^204^Hg and MM^200^Hg were added to the water to concentrations of 3.3–7.8 and 2.3–7.6 ng L^–1^, respectively. Prior to the experiment, DM^204^Hg and MM^200^Hg were allowed to equilibrate with the water in the dark for 2 h. The incubations were thereafter initiated after ∼1 h (during the setup, the flasks were stored under ambient laboratory light). The headspace was always kept small (ca 1 mL) to avoid loss of gaseous Hg species during the experiment. Flasks were oriented with the flat surface facing the source of light. Except for experiments j, m, and n (Supporting Information Table S1), samples were prepared in triplicates. Experiments probing the effect of different wavelength spans (experiments m and n, Supporting Information Table S1) were performed using a longer flask (220 mm) with curved surfaces. For these experiments, a cassette was used, where the light was only let in through an opening that could be fitted with different filters. These experiments were executed with DMHg and MMHg of natural isotopic abundance of Hg, hereafter referred to as^amb^Hg, and added in separate incubations without equilibration times.

Indoor experiments exploring photodecomposition of DM^204^Hg and MM^200^Hg in the different waters (experiment a–d, Supporting Information Table S1) were all subsampled after 0, 30, 75, 150, and 240 min of exposure (Supporting Information Table S1). When subsampling, 4.6–9.7 mL of the sample was extracted with a pipette and injected under the water surface into a 40 mL vial with Milli-Q, acetate buffer, and 1–2 ppt of isotopically enriched ^199^Hg and MM^200^Hg (internal standards to quantify Hg^II^ and MMHg). The experimental solution was refilled with the same amount of corresponding sample water removed during the subsampling and then quickly closed. A DM^amb^Hg standard was thereafter added to the subsamples with a Hamilton syringe to a final concentration of ∼1 to 2 ppt as an internal standard for DMHg. An amount of 30 μl of 1% NaTEB in 2% KOH was added to each subsample, which was then closed and shaken. Previous analysis with and without NaTEB and buffer has demonstrated DMHg to be stable during ethylation (Supporting Information Figure S2). The total volume of subsamples was 30 mL. Samples were left to react for at least 15 min before the analysis.

Natural sunlight experiments were performed at the Stockholm University campus (59°21N, 18°03E) on two mostly cloud-free days in July 2021. Incubation flasks were covered with black tape on all sides except the flat top and placed horizontally on an open grass field without shadow-casting objects. The exposed waters were subsampled before and after the exposure period. The temperature was continually measured by a thermometer placed next to the cylinders, and it was confirmed that the temperature did not exceed 40 °C. Light intensity data collected from a nearby (∼100 m) weather tower was provided by the Department of Meteorology (Stockholm University) and complemented with handheld UV measurements in close proximity to the samples.

Dark control experiments were performed for all water types in a temperature-controlled water bath at 40 °C for 6 h. Subsamples were collected before and after the incubation.

### Analytical Methods

Hg^II^, MMHg, and DMHg were quantified using a Tekran 2700 methylmercury analyzer coupled to an inductively coupled plasma-mass spectrometer (ICP-MS, Thermo Scientific iCAP Qnova series). Before the samples were purged, ionic forms of Hg (Hg^II^ and MMHg) were ethylated using NaTEB (as described above). The Tekran 2700 analyzer was used to preconcentrate and separate the different forms of Hg. The two instruments were coupled using Teflon tubing from the sample outlet on the methylmercury analyzer to the ICP-MS. All forms of Hg were combusted to Hg^0^ in the methylmercury analyzer before leaving the sample outlet, and thus, a heated transfer line was not required.

The Tekran 2700 system was calibrated daily and used to quantify the stock solutions of ^199^Hg^II^, MM^200^Hg, MM^201^Hg, DM^204^Hg, and DM^amb^Hg. The average relative standard deviation (RSD%) of 4–5 replicate sample analyses of ^199^Hg, MM^200^Hg, MM^201^Hg, and DM^204^Hg was 5.2%, whereas three separate analyses of ambient DMHg injections had an RSD% of 2.1% (Supporting Information Figure S3). While the analytical setup used also isolated and detected Hg^0^, Hg^0^ was only present in low quantities (<4% of initially added MM^200^Hg or DM^204^Hg) and thus not discussed further. Analysis of MMHg stock dilutions (*n* = 8, not isotopically enriched) revealed good precision within the relevant concentration range as well as a low mass bias effect.

Organic carbon content (measured as dissolved or the total concentrations depending on whether the waters used for the incubations were filtered or not prior to the incubation) was measured with a total organic carbon analyzer (TOC-L, Shimadzu, Japan). Briefly, 30 mL of the sample was transferred into 40 mL glass vials closed with septa caps. All samples and blanks were acidified with HCL to 0.5% v-v and measured in triplicates. The OC-rich streamwater sample was diluted by a factor of 30, 10, 3, and 2 with Milli-Q water before the analysis.

### Data Processing

All Hg species concentrations, except for DMHg concentrations in experiment a (SI Table S1) were quantified from internal ^199^Hg, MM^201^Hg, and DM^amb^Hg standards through signal deconvolution.^10^

Photodecomposition was assumed to follow pseudo-first-order kinetics, and decomposition rate constants (*k*_d_) were calculated according to eqs 1 and 2(11)
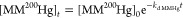
1
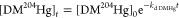
2

For lamp exposure experiments, demethylation rates were calculated for 75 min of exposure time. For outdoor incubations, demethylation rates were calculated for the end of the experiment (4.5 and 8 h for experiments e and f, respectively).

Statistical treatment of the data was done using JMP Pro (version 15.0.0) statistical software. Normality of the data distribution was tested using Shapiro–Wilk normality test and by visually inspecting the density plots and Q–Q plots. To examine if there were differences between sample groups, the normal (or log-normal) data was tested using a one-way ANOVA. Comparison between groups was then done using Tukey’s pairwise post-hoc analysis. Changes in concentrations during experiment f were tested with paired Student’s *t*-test.

## Results and Discussion

### Photochemical Degradation of DMHg

**Figure 1:**
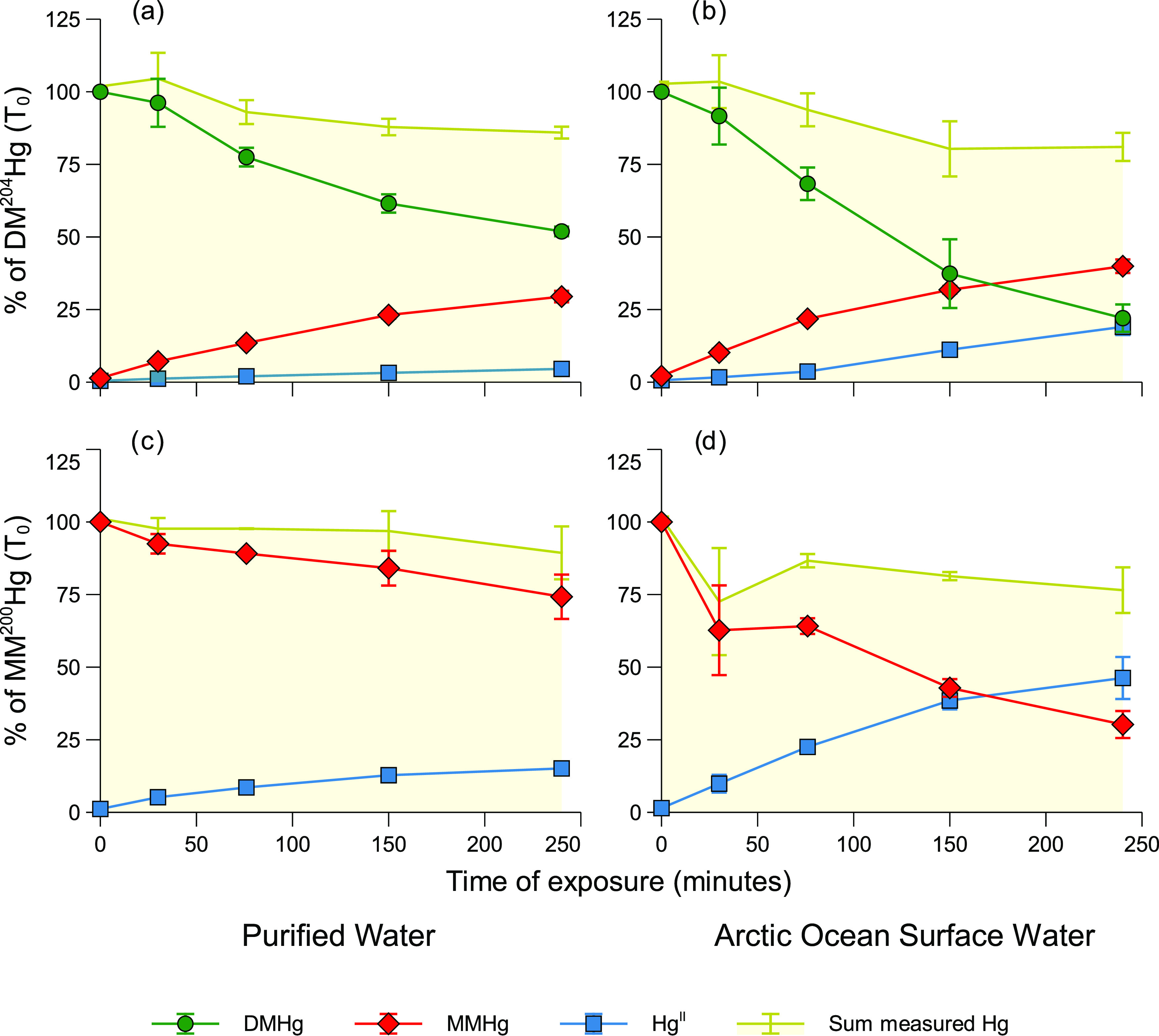
Changes in DM^204^Hg and MM^200^Hg concentrations and corresponding photodecomposition products over time for experiment d (Supporting Information Table S1). Photodecomposition of DM^204^Hg in (a) purified water and (b) Arctic Ocean surface water, and photodecomposition of MM^200^Hg in (c) purified water and (d) Arctic Ocean surface water. The sum of measured Hg species includes DM^204^Hg + MM^204^Hg + ^204^Hg^II^ for panels (a) and (b) and MM^200^Hg + ^200^Hg^II^ for panels (c) and (d). Error bars represent one standard deviation of triplicate incubations.

To test whether or not DMHg in natural waters is subjected to photochemical degradation, the stability of an isotopically enriched DMHg tracer (DM^204^Hg) dissolved in water was tested under artificial UV radiation. The concentrations of DM^204^Hg decreased during the light exposure in all of the tested waters (Figure 1 and Supporting Information Figures S4–S6). The concurrent increase of MM^204^Hg shows that the loss of DM^204^Hg observed was a result of degradation (demethylation) rather than evasion or adsorption of DM^204^Hg to the walls of the reaction flasks. The stability of added DM^204^Hg in control experiments incubated in the dark at 40 °C (Supporting Information Figure S7) further confirms that the decomposition observed under light was photochemically mediated.

While photochemical degradation of DMHg in waters has been previously debated,^3,5^ photochemical degradation of MMHg has been shown in a range of different waters and is widely acknowledged as an important Hg demethylation pathway in sunlit waters.^12−20^ In all of our experiments utilizing isotopic tracer techniques (experiments a-l, Supporting Information Table S1) a second isotopically enriched tracer (MM^200^Hg) was added to simultaneously study the decomposition of MMHg. Using previously reported demethylation rates of MMHg as a reference point, this allows us to further evaluate the relevance of DMHg photodegradation in natural systems by comparing the demethylation rates of DM^204^Hg to those of MM^200^Hg in our experiments. Degradation of MMHg was observed in the purified water, Arctic, and Baltic surface water samples, as well as in the artificial seawater. An increase in inorganic divalent Hg (^200^Hg^II^) over the course of the incubation also confirmed the photochemical demethylation of MM^200^Hg in the streamwater (Figure 1, Supporting Information Figures S4–S6). As for the DM^204^Hg tracer, no degradation of MM^200^Hg was observed in the dark control experiments (SI Figure S7).

In all experiments, DM^204^Hg decomposition was mainly coupled to the formation of MM^204^Hg (Figure 1 and Supporting Information Figures S4–S6). With time, MM^204^Hg concentrations typically plateaued, and concentrations of ^204^Hg^II^ increased. The mass balance for ^204^Hg observed during the experiments (DM^204^Hg+MM^204^Hg+^204^Hg^II^) ranged from 80 to 105%, with the exception of experiment a, where the recovery ranged up to 139% during the experiment (Supporting Information Figure S4). For the MM^200^Hg tracer, 57–117% of the initially added MMHg was accounted for as MM^200^Hg or ^200^Hg^II^. Only for the Baltic waters was this recovery below 70%. To conclude, we show that the full demethylation of DMHg is a two-step process, where DMHg is first degraded to MMHg, which is then further demethylated to Hg^II^.

### Degradation of DMHg in Purified Water

**Figure 2:**
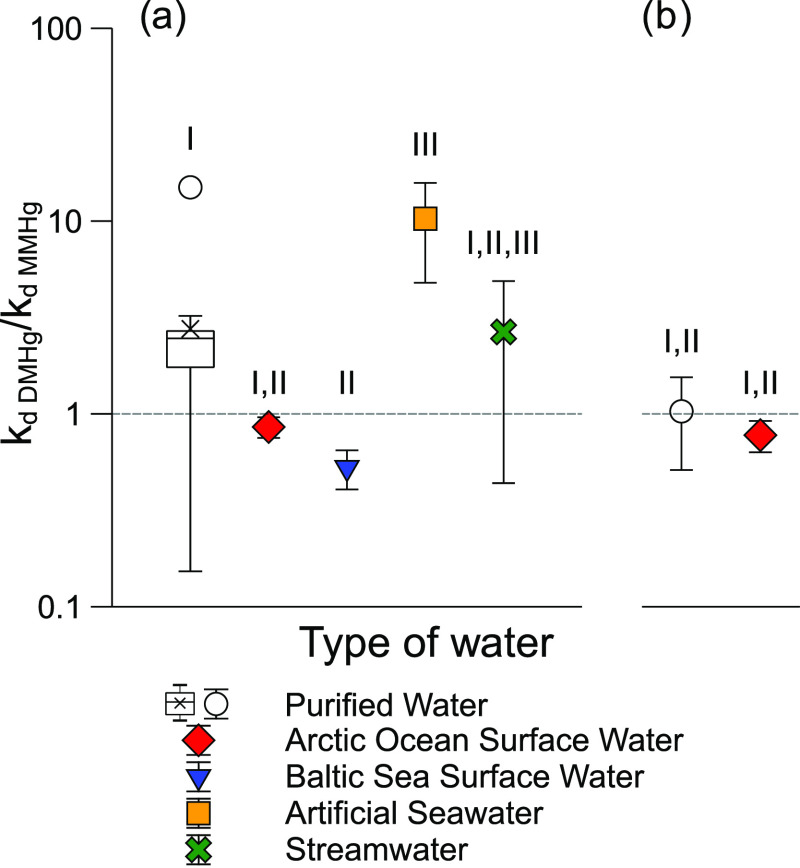
The *k*_d DMHg_ to *k*_d MMHg_ ratio in tested waters for (a) UV lamp and (b) sunlight experiments. The dashed line indicates a ratio of 1. UV lamp exposure incubation with purified water (box plot) was replicated 19 times. Whiskers in the box plot show 1.5*IQR. For all other sample groups, *n* = 3, and error bars show one standard deviation of replicate incubations. Roman numbers indicate significant differences (*p* < 0.05).

The stability of DM^204^Hg (and MM^200^Hg) in purified water (Milli-Q) was tested in several experiments where the waters were exposed to artificial or natural sunlight (Supporting Information Table S1). Although degradation of both DM^204^Hg and MM^200^Hg in the purified water was observed in all of the experiments using the UV lamp, the rate of photodegradation differed between the experiments. In addition, relatively large variations in the amounts of DM^204^Hg, MM^200^Hg, and the degradation products of both tracers were observed in experiments a and b (Supporting Information Figures S4 and S5). These variations could be explained by differences in reaction rates (Supporting Information Figures S8 and S9). The Osram HTC 400-241 lamp, which was mounted inside the fume hood under a stream of air to prevent overheating, was chosen due to its agreement with solar spectral irradiance at surface level in the UV light range (Figure S1). Even though the samples were placed in a circle at the same distance from the lamp, variations in the radiation based on angular location were noted and could potentially explain the large variation between the replicates in experiments a and b. It should however be noted that the relative variation between triplicate samples in the other experiments was within a few percent, suggesting the angular location of the reaction vessels to not be critical for the rates determined in these cases. The aging of the lamps used and daily variabilities could, however, contribute to the variations observed for purified water between experiments. Assuming no change in characteristics of the emitted light, the ratio of *k*_d DMHg_ to *k*_d MMHg_ should however not be affected as the degradation rate of DMHg and MMHg were quantified simultaneously using tracers enriched in different isotopes. For purified water, the *k*_d DMHg_ to *k*_d MMHg_ ratio was ∼2.5, suggesting that DMHg degrades faster than MMHg (Figure 2). Photodecomposition of MMHg in purified water has been previously examined, with some studies reporting decomposition^14,18^ and others not doing so.^13,17,19,21^ It is possible that small differences in the purity of the water caused the observed difference between studies and also between experiments in our study. The photochemical degradation of MMHg has previously been closely linked to DOC concentrations and characteristics, specifically, the availability and type of thiol-containing compounds.^19,22−24^ Since Milli-Q contains DOC in ppb-range concentrations, variation in concentration or availability of binding sites for MMHg in the purified water could possibly account for the differences observed in our study as well as for the observations of others.

### Degradation of DMHg in Natural Waters

In addition to the purified water, photochemical degradation of DM^204^Hg (and MM^200^Hg) using the UV lamp was also tested in artificial seawater and surface waters collected from the Arctic Ocean, the Baltic Sea, and a forest stream. As mentioned above, the aging of the lamps used and daily variability in the radiation produced, resulting in a variation in the demethylation rates of both methylated forms in purified water, prevents direct comparison of the rates generated between the experiments. For the purpose of enabling comparison between samples, it is assumed that the variations in lamp irradiation did not alter the *k*_d DMHg_ to *k*_d MMHg_ ratio. These ratios were thus used to (i) explore differences in Hg demethylation processes between the water types tested and (ii) to evaluate the importance of DMHg demethylation rates in natural waters (as there are data on MMHg demethylation rates available from previous studies for comparison).

The highest average *k*_d DMHg_ to *k*_d MMHg_ ratio was observed in the artificial seawater (Figure 2; *k*_d DMHg_ to *k*_d MMHg_ ratio for artificial seawater greater than *k*_d DMHg_ to *k*_d MMHg_ ratios for purified water, Arctic Ocean, and Baltic surface waters (*p* < 0.05); *k*_d DMHg_ to *k*_d MMHg_ ratio for artificial seawater = *k*_d DMHg_ to *k*_d MMHg_ ratio for DOM (*p* > 0.05)). The observed *k*_d DMHg_ to *k*_d MMHg_ ratio for the Baltic water was lower than the ratio observed in purified water (*p* < 0.05). In all of the experiments, the stability of DMHg (and MMHg) was tested in purified water in parallel to the natural waters. Although we chose not to use it as an approach to directly compare the demethylation rates between the waters, we calculated decomposition rate constants for the natural waters normalized after purified water to examine if the differences observed in the *k*_d DMHg_ to *k*_d MMHg_ ratio were driven by changes in the demethylation rate of DMHg or MMHg. The comparison of the purified water-normalized *k*_d_ values suggests that the high *k*_d DMHg_ to *k*_d MMHg_ ratio observed for the artificial seawater is due to lower demethylation rates of MMHg in the artificial seawater in comparison to the other waters tested (*p* < 0.05). The artificial seawater was also the water that had the lowest DOC content (0.9 mg L^–1^ in comparison to the other waters, where the DOC ranged from 1.3 up to 75 mg L^–1^, Supporting Information Table S2). To further examine the effect of DOC content on the DMHg and MMHg photodemetylation rates, we performed an incubation where the artificial seawater was mixed with the DOC-rich streamwater, resulting in DOC concentrations ranging from 0.9 to 2.4 mg L^–1^ (Supporting Information Figure S10). MMHg decomposition (observed by the formation of Hg^II^) was only detectable in the mixture with the highest DOC content (2.4 mg L^–1^) but not in the other mixtures (DOC ranging from 0.9 to 1.3 mg L^–1^). In contrast, DMHg demethylation rates did not differ between the waters and thus further support the fact that DOC facilitates the photochemical degradation of MMHg, but it does not affect the photochemical degradation rate of DMHg in the DOC concentrations tested. The role of DOC in the photochemical demethylation of MMHg has previously been investigated. At higher DOC concentrations, lower demethylation has been observed as DOC reduces light attenuation. At the same time, DOC may absorb light and act as a precursor for radicals important for the demethylation of MMHg.^15,16,23^ The binding of MMHg to sulfur-containing organic ligands has also been suggested to make MMHg more susceptible to photochemical degradation due to the lower excitation energy of the Hg-C bond (in contrast to when it is complexed to Cl).^23,25^ If assuming a similar concentration of thiols in the artificial seawater DOC as what has previously been shown for natural OC, the thermodynamic speciation model (SI discussion) suggests over 99% of the MMHg to be complexed with DOC. The thermodynamic speciation model further suggested the same to be true for the natural waters tested as well as for the artificial seawater and DOC mixtures prepared. The chemical speciation of MMHg thus suggests that the differences observed cannot be explained by what complexing ligand MMHg is bound to. These results are in line with an earlier study where salinity was concluded to affect the demethylation rates of MMHg even for sets of waters, where MMHg was mainly complexed to DOC.^26^ As DMHg is fully methylated and appears as a dissolved gas, complexing ligands are less likely to play a role in the demethylation potential. It should however be noted that DMHg recently was demonstrated to degrade in the presence of dissolved sulfide and mackinawite (FeS(s)),^8^ and thus the role of potential complexing ligands cannot simply be ignored. Additionally, the complexation of MMHg by DOC could possibly have an indirect effect on DMHg photodecomposition rates by inhibiting any potential remethylation of MMHg to DMHg. Finally, reduced light attenuation through absorption of photons by chromophoric DOC is likely to influence rates of photodecomposition for both DMHg and MMHg.^18^ For the Baltic water, the purified water-normalized k_d_ values suggest that the low *k*_d DMHg_ to *k*_d MMHg_ ratio observed was due to lower demethylation rates of DMHg (*p* < 0.05). The reason behind this is less clear as the Baltic water had intermediate concentrations of DOC and Cl. Although we cannot explain the differences observed, and our discussion relies on the ratio of *k*_d DMHg_ to *k*_d MMHg_ to remain unchanged between experiments, it is interesting to note that our comparison hints toward differences in the photochemical degradation pathways of DMHg contra MMHg.

### Degradation of DMHg When Exposed to Different Wavelength Regimes

Studies on MMHg photodecomposition have previously shown that MMHg photodecomposition efficiency varies by 2–3 orders of magnitude depending on the wavelength, where MMHg is most effectively degraded under UVB (280–315 nm), followed by UVA (315–400 nm), and finally PAR (400–700 nm).^14,15^ These observations have been explained by the formation of reactive radical species (e.g., OH^–^, singlet oxygen (^1^O_2_), and triplet-state photosensitized natural organic matter (NOM^3^).^13,21^ Direct photodecomposition through the absorption of light by MMHg (not accounting for ligands it may be complexed to) as well as DMHg is unlikely to have environmental significance, as these molecules primarily absorb light in the UVC wavelength region (which is absent in the sunlight reaching surface waters^27^).^9^ In preliminary experiments with the UV lamp (experiments m and n, Supporting Information Table S1), rates of DMHg photodecomposition increased dramatically when the water was exposed to wavelengths below 305 nm (by removing the filter). A similar effect was also observed for MMHg (Supporting Information Figure S11). We also tested the relative importance of UVB and UVA for DMHg decomposition. This was done by replacing the 305 nm filters with 320 nm filters for one purified water sample in experiments k and l (Supporting Information Figure S12, Supporting Information Table S1). While the results were unclear for MMHg due to large variation in MMHg demethylation rates, DMHg decomposition was consistently slower when radiation with wavelengths of 305–320 nm was blocked (Supporting Information Figure S12a)). The reduction in DMHg demethylation rate was greater than the reduction of light intensity with the 320 nm filter (Supporting Information Table S3, Supporting Information Figure S12b), suggesting that UVB more effectively demethylates DMHg when compared to UVA (in line with what has previously been shown for MMHg^15,28^).

### Outdoor Experiments

**Figure 3:**
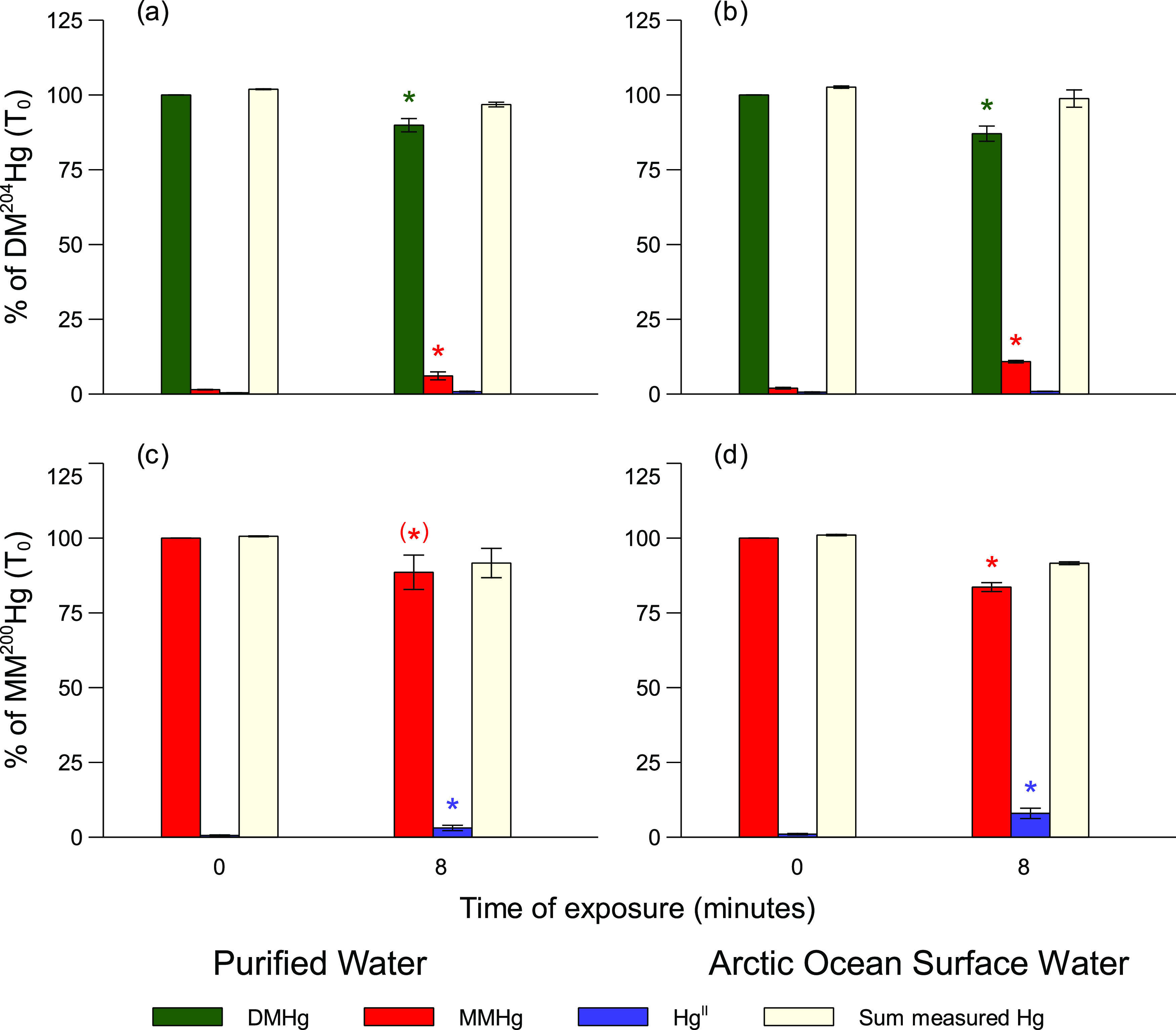
Changes in DM^204^Hg and MM^200^Hg concentrations and photodecomposition products between initiation and termination of the outdoor experiment (experiment f, Supporting Information Table S1). Photodecomposition of DM^204^Hg in (a) purified water and (b) Arctic Ocean surface water, and photodecomposition of MM^200^Hg in (c) purified water and (d) Arctic Ocean surface water. The sum of measured Hg species includes DM^204^Hg + MM^204^Hg + ^204^HgII for panels (a) and (b) and MM^200^Hg + ^200^Hg^II^ for panels (c) and (d). Error bars represent one standard deviation of triplicate incubations. Asterisks show statistically significant differences in concentrations between T0 and T1 (*p* < 0.05). The decrease in MM^200^Hg in purified water (marked by “(*)” in panel c)) was statistically significant at the *p* < 0.1 level (*p* = 0.075).

To test whether or not DMHg is also decomposed when exposed to natural sunlight, purified water and Arctic surface water amended with the DMHg and MMHg tracers were incubated outdoors. In the longer, 8-h experiment (experiment g, Supporting Information Table S1), we noted degradation of the DM^204^Hg tracer and formation of MM^204^Hg as a degradation product (*p* < 0.05). Although no significant decrease in MM^200^Hg (*p* = 0.075) was observed, concentrations of ^200^Hg^II^ increased, suggesting that also the MM^200^Hg was demethylated (Figure 3). These results were used to calculate *k*_d MMHg_ and *k*_d DMHg_ for the incubated waters and could be related to the light intensity measured over the course of the incubations (Table 1, Supporting Information Figure S13). In a similar but shorter outdoor experiment (experiment f, 4.5 h, Supporting Information Table S1), no significant loss of DM^204^Hg or MM^200^Hg was observed. A higher concentration of MM^204^Hg after the incubation (*p* = 0.0004), however, shows that degradation of the dissolved DM^204^Hg tracer had occurred. The *k*_d DMHg_ to *k*_d MMHg_ ratios for the purified water and the Arctic surface water from the outdoor experiments were in good agreement with the ratios obtained from the laboratory experiments using a UV lamp (Figure 2). In addition, MMHg demethylation rates calculated for experiment g (0.37–0.54 d^–1^, Table 1) were also in good agreement with previously measured rates of MMHg under natural sunlight (0.03–1.67 d^–1^).^18^ To conclude, our outdoor experiments confirm the results from our indoor experiments that DMHg, like MMHg, is photochemically degradable in natural light.

**Table 1:** Summary of Geometrical Parameters, DMHg and MMHg Initial Concentrations and Photodemethylation Rates, and Light Intensity Data for Experiment f; Incubations with Natural Sunlight

	purified water	Arctic surface water
exposure area	19.6 × 10^–4^ m^2^	19.6 × 10^–4^ m^2^
sample volume (mL)	114.1 ± 1	114.5 ± 2.3
MMHg initial concentration (pM)	22.8 ± 0.7	21.1 ± 0.8
DMHg initial concentration (pM)	20.6 ± 0.4	20.6 ± 0.6
*k*_d MMHg_ (d^–1^)	0.37 ± 0.2	0.54 ± 0.05
*k*_d DMHg_ (d^–1^)	0.32 ± 0.07	0.42 ± 0.09
average light intensity (W m^–2^)	581.7	581.7
total light intensity (Whr)	9.13	9.13

Our experimental setup required us to add DM^204^Hg and MM^200^Hg in concentrations higher than those of natural waters. This means that the relative proportions of the reactants (and possibly also the aqueous speciation of MMHg) in our experiments may differ from natural conditions. Our MMHg decomposition rates (from our outdoor experiments) are, however, comparable with those observed in previous studies (also including studies done at lower MMHg concentrations).^18^ For DMHg, the ratio between DMHg and photoreactants responsible for the photodemethylation observed could potentially play a role. If assuming that the amounts of photoreactants produced would limit the demethylation of DMHg, we would expect the rates to increase at lower DMHg concentrations. It is therefore more likely that we here underestimate, rather than overestimate, the degradation rates of DMHg in natural waters.

## Environmental Implications

DMHg appears to be ubiquitous in marine waters, where it has been reported at concentrations ranging from 0.01 to 0.4 pM. The vertical profile of DMHg in marine waters typically resembles the vertical distribution of MMHg, with lower concentrations in surface waters and the greatest concentrations in the organic matter remineralization zone and deeper waters. While concentrations of DMHg in the deeper ocean waters often exceed those of MMHg, MMHg is typically the main methylated form of Hg in surface waters. The low concentrations of DMHg in surface waters have previously been assumed to be caused by the evasion of DMHg to the atmosphere.^29−32^ Although MMHg is commonly assumed to be the primary methylated form of Hg in freshwater systems, DMHg has also been reported from, e.g., lakes, floodwaters, and freshwater sediments.^33−36^ It should also be noted that only a few studies have aimed to quantify DMHg in such systems. Instead, MMHg is typically measured in samples preserved by acidification, which also is known to degrade DMHg to MMHg.^5^ Recent studies have also reported DMHg in the brackish Baltic Sea, although these studies suggest that DMHg in these waters composes a smaller fraction of the methylated pool than in marine systems.^37,38^ Here, we resolve contradictory claims in the literature and can show that DMHg is readily degraded under light when dissolved in both marine and brackish surface water as well as in the freshwater collected. Although photochemical degradation of DMHg in fresh and brackish waters is less likely to be an important source of MMHg, it could still be a process contributing to the lower DMHg concentrations found in these environments. This inference is supported by the relatively high *k*_d DMHg_ to *k*_d MMHg_ ratios observed for the streamwater sample and by the fact that photochemical demethylation of MMHg is recognized as an important degradation pathway of MMHg in sunlit waters.

For the Arctic Ocean waters that also was incubated under natural sunlight, we observed a decomposition rate (0.42 d^–1^, Table 1) that is just below the k_d DMHg_ previously reported from incubations with Pacific seawater (0.52–1.64 d^–1^).^3^ It should, however, be noted that the previously reported constants likely overestimate the photochemical demethylation rates of DMHg as the losses from dark controls (0.16–0.22 d^–1^, e.g., due to evasion of DMHg through the Teflon flasks used) were not accounted for. Black et al.^5^ argued DMHg to be stable in surface waters and the rate of potential demethylation of dimethylmercury in surface waters to not exceed the estimated evasion fluxes of DMHg to the atmosphere. Our study presents photodemethylation rates that are up to 40 times faster than the evasion fluxes calculated by Black et al., indicating that DMHg transferred from deeper waters by advection to marine surface waters may be photochemically degraded under light hours rather than lost through evasion. The discrepancy between the results by Black et al. and this study could be partly explained by differences in light transmission between the borosilicate bottles used in the former study and the quartz bottles used here. While the borosilicate bottles used by Black et al. have a generally lower light transmission throughout the visible light spectrum, this transmission is much lower in the UVB spectrum range, which our data indicate is important for DMHg decomposition.^5^

Given the similar *k*_d_ values observed for MMHg and DMHg in the Arctic Ocean water incubated (under natural sunlight) and that photochemical demethylation of MMHg is recognized as an important degradation pathway of MMHg in marine surface waters, we propose that photochemical demethylation of DMHg in sunlit seawater could be an important source of MMHg. To further examine this, we calculated the potential flux of DMHg to MMHg using the *k*_d DMHg_ to *k*_d MMHg_ ratio we observed for the Arctic Ocean water together with the MMHg → Hg^II^ flux (31 Mmol yr^–1^) and the size of the MMHg and DMHg pools (1.5 Mmol and 0.36 Mmol, respectively) proposed for the upper 100 m in a recent global marine mass budget for Hg.^32^ By assuming a similar relationship between the MMHg → Hg^II^ flux and the MMHg pool to be between the DMHg → MMHg flux and the DMHg pool (but *k*_d DMHg_ = 0.8 *k*_d MMHg_), we get a DMHg → MMHg flux of 6 Mmol yr^–1^ (DMHg → MMHg flux (Mmol yr^–1^) = (0.8 · 31 Mmol yr^–1^ · 0.36 Mmol) · (1.5 Mmol)^−1^). As a comparison, the Hg^II^ → MMHg flux (due to in situ methylation) estimated in the global mass balance was estimated to 28 Mmol yr^–1^.^32^ Although the DMHg → MMHg flux could be limited by the advection rate of DMHg from deeper waters, this comparison implies that DMHg photodecomposition could represent a significant source of MMHg in surface oceans that then could be available for bioaccumulation in the food web through the uptake of MMHg by primary producers.

## Supporting Information Available

Synthetization of DMHg standard; MMHg speciation model; summary of the experiments (Table S1); OC concentrations and pH (Table S2); wavelength spectra for natural sunlight and UV lamp, light absorbance of MMHg and DMHg, and light transmission of quartz glass and filters (Figure S1); test on the effect of buffer and ethylation agent for MMHg analysis on DMHg recovery and stability (Figure S2); comparison when subsampling with a syringe or pipette (Figure S3); degradation of DMHg and MMHg in contrasting waters (Figures S4–S6), in dark controls (Figure S7), purified, Baltic and Arctic Ocean water shown for each replicate (Figures S8 and S9), in artificial seawater with different concentrations of DOC (Figure S9), with and without filter (Figure S11) and under different wavelength regimes (Figure S12); light intensity during the outdoor experiment (Figure S13), DMHg degradation in experiment with excess of buffer (Figure S14) (PDF)
