# Supplementary material for: Photochemical Degradation of Dimethylmercury in Natural Waters
Source: Environ Sci Technol. 2022 Apr 20;56(9):5920–8. doi: 10.1021/acs.est.1c08443 (PMC9069699; doi:10.1021/acs.est.1c08443)
Supplement: Supplementary file 1 — es1c08443_si_001.pdf [file es1c08443_si_001.pdf]

Supporting Information

Photochemical Degradation of Dimethylmercury in Natural Waters

Johannes West[1], Sonja Gindorf[1], Sofi Jonsson[1]*

[1]Department of Environmental Science, Stockholm University, 106 91 Stockholm, Sweden
*Corresponding Author: sofi.jonsson@aces.su.se

Supporting information contains 2 tables, and 13 figures

Index

Synthetization method for a DMHg standard free from THF S1

MMeHg speciation model S1

Table S1 S2

Table S2 S3

Figure S1-14 S4-S21

References S22

Synthetization method for a DMHg standard free from THF

The DMHg standard was prepared in a 40 ml amber glass vial sealed with septa and filled with Milli-Q water and 0.75% 2 M acetate buffer. A 4 ml amber glass vial (hereon referred to as the reaction vial) was inserted into the 40 ml vial. The reaction vial was placed on a stand so that the buffered Milli-Q water in the 40 ml vial did not flow into it. In the reaction vial, 7 ul 1000 mg L^{-1} Hg^{II} in 12% v/v HNO_3 (AAS standard, Sigma Aldrich) was mixed with methylcobalamin (MeB12), a methyl donor previously used for DMHg synthetization,[1–3] to a Hg:MeB12 ratio of 1:50. The reaction solution was buffered to pH 5 using acetate buffer to a total volume of 1 ml. The reaction was allowed to continue overnight (~17 hrs) double-wrapped in aluminum foil. DMHg formed from the reaction preferentially diffused into the larger volume and could be collected using a syringe with a needle through the septa. A $DM^{204}Hg$ stock solution was prepared similarly by adding isotopically enriched $^{204}Hg^{II}$ (CortecNet), HNO_3, MeB12, and acetate buffer to the reaction vial in the same proportions as above. The total yield of collected DMHg in the buffered Milli-Q water was ~14-24% of originally added Hg^{II}. These yields are comparable to previously reported yields of DMHg when reacting Hg^{II} with MeB12 under similar conditions.[4] As MMHg and Hg^{II} are not volatile forms of Hg, the purity of the standard was high (< 1 % MMHg and < 1% HgII). Control experiments (SI Figure S14), where an excess of the buffer was added to purified water, confirmed that the small amounts of acetate buffer that was added to the experiments with the standard did not result in altered decomposition rates of DMHg.

MMHg speciation model

The speciation of MMHg was modeled using the thermodynamic formation constants (Log K) of 5.4 for MMHgCl,[5] and 17.5 for MMHg-SRDOC (aq)[6] together with the concentrations of thiols (calculated as 0.15 % * DOC), MMHg, and Cl[7,8].

Table S1. Summary of experiments. Type of radiation, type, and age of water, as well as initial DMHg and MMHg concentrations, are listed.

Experiment	Radiation source	Water types tested	Age of natural water (days)[1]*	DM204Hg initial concentration[2] ng L^{-1}	MM200Hg initial concentration[2] ng L^{-1}	Description
a	UV lamp	Purified water, Baltic Sea surface water	6	4.8 [3]	6.0	
b	UV lamp	Purified water, Artificial seawater (30.7‰)		7.5	5.1	
c	UV lamp	Purified water, Streamwater	1	5.8	4.3	
d	UV lamp	Purified water, Arctic Ocean surface water	9	5.2	4.9	
e	Outdoor	Purified water, Arctic Ocean surface water	3	6.3	4.9	
f	Outdoor	Purified water, Arctic Ocean surface water	16	4.1	4.4	
g	Dark	Baltic Sea surface water, Arctic Ocean surface water	22 (Baltic), 10 (Arctic)	6.4	4.1	Dark control experiment
h	Dark	Purified water, Streamwater	5	6.1	3.3	Dark control experiment
i	Dark	Artificial seawater (29.2‰)		3.4	3.8	Dark control experiment
j	UV lamp	Artificial seawater (29.2‰), Streamwater	12	6.9	4.1	Investigating effect of increasing DOC.
k[4]	UV lamp	Purified water		5.2	4.4	
l[4]	UV lamp	Purified water		4.1	4.4	
m[5]	UV lamp	Purified water		5.6		Effect of filter removal on DMHg photodemethylation.
n[5]	UV lamp	Purified water			5.8	Effect of filter removal on MMHg photodemethylation

[1] Number of days of water stored at 4°C after initial preparation (Instant ocean salt solution)/thawing (Arctic surface waters)/collection (DOC-rich freshwater+Baltic surface waters)

[2] Averaged from all measurements at T_0

[3] DMHg quantified from T0 response factor based on stock concentrations measured the day before

[4] For one sample container in each experiment, one 305 nm cutoff filter was exchanged for a 320 nm cutoff filter

[5] Experiments were conducted using 220ml long flasks, mounted in cassettes. only ambient DMHg and MMHg was used in the experiments

Table S2. OC concentrations and pH for the various water types. For OC analysis, the limit of detection (~0.17 mg L^{-1}) and limit of quantification (~0.55 mg L^{-1}) were calculated as the threefold and the tenfold of the standard deviation of Blanks (n=51), respectively.

**Sample**	**OC [mg L^{-1}]**	**OC STD**	**pH**
Artificial seawater (filtered 0.2µm), 29.2‰	0.90	0.30	9.5
Arctic Ocean surface water (unfiltered)	1.4	0.033	8.6
Baltic Sea surface water (unfiltered)	4.0	0.054	8.4
Streamwater (filtered 0.45µm)	75	1.8	6.6

Table S3. UV Light intensity measurements performed at a fixed angle and various intervals from the UV lamp, with 305 and 320 nm cutoff filters mounted on the sensor, respectively. Samples were spaced approximately 12 cm from the light source.

Distance (cm)	µW cm^{-2} λ> 305nm	µW cm^{-2} λ> 320nm
60	106	75
50	179	121
40	303	193
30	402	280
20	817	642
15	1498	1048

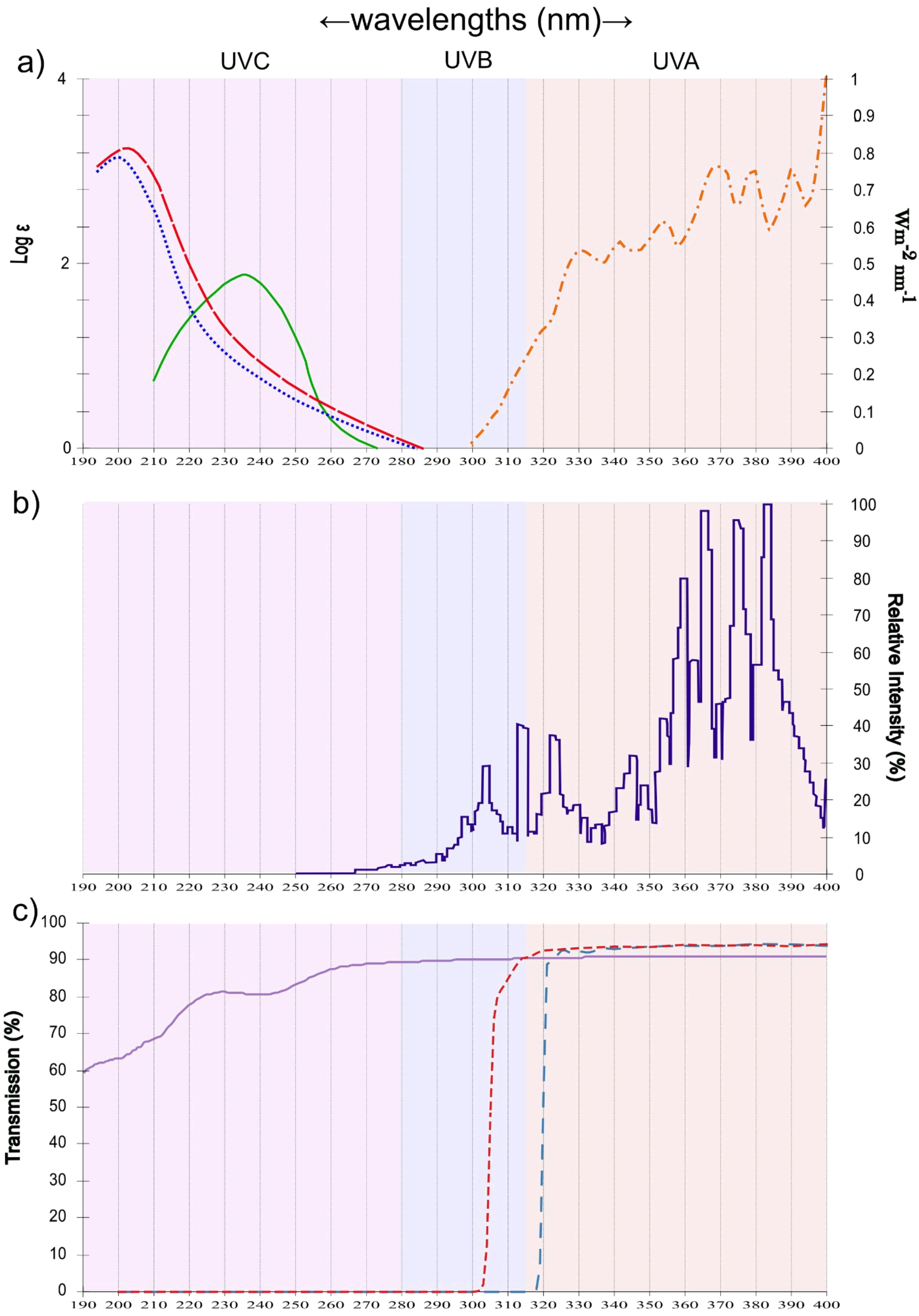
←wavelengths (nm)→
a)
UVC
UVB
UVA
Log ε
Wm-2 nm-1
b)
Relative Intensity (%)
c)
Transmission (%)
190 200 210 220 230 240 250 260 270 280 290 300 310 320 330 340 350 360 370 380 390 400

Figure S1. summary of wavelength-dependent factors in the UV spectrum, with the light regimes of UVA, UVB and UVC marked out as shaded areas. a) Measured absorption spectra (Log ε) of DMHg at pH 7 (green solid line), $MMHg^+$ at pH 2 (red dashed line), and MMHgOH at pH 10 (blue dotted line)[9], together with the solar spectral irradiance ($Wm^{-2}nm^{-1}$) measured at noon, clear days at 38°N.[10] b) Spectral radial distribution of OSRAM 400-241 UV lamps c) The light transmission in % for 2mm thick Ilmasil PN quartz glass (solid purple line), Newport 20CGA-305 filters (red thinly dashed line) and Newport 20CGA-320 filters (blue thickly dashed line).

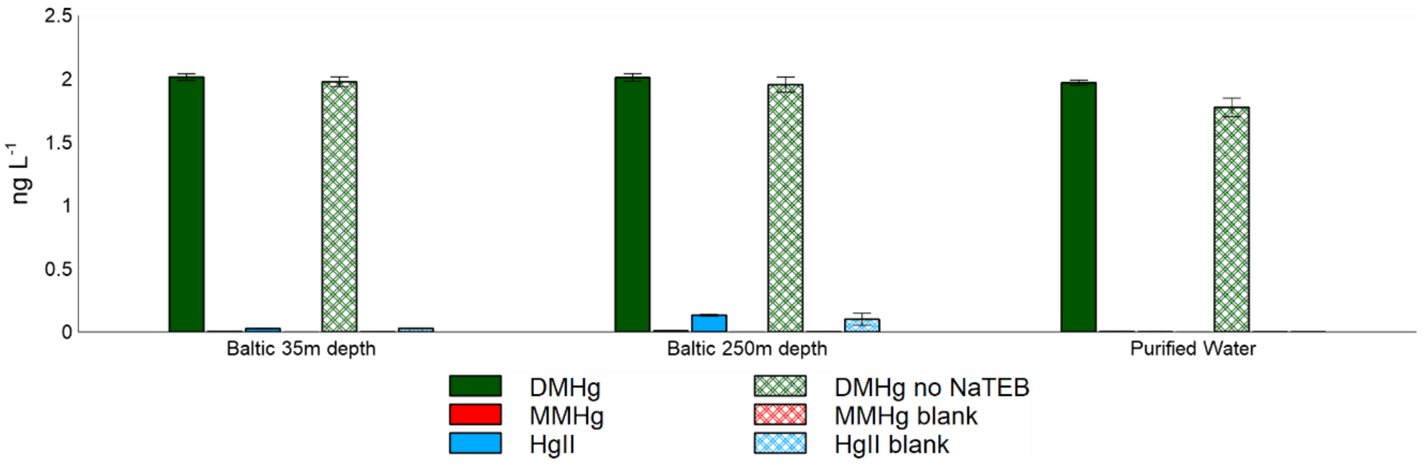

Figure S2: The effect of acetate buffer and sodium tetraethyl borate (NaTEB) on DMHg recovery and stability during DMHg analysis. The Baltic waters were collected from Landsort deep on the 16th of March 2020 and stored refrigerated until experiment on 9th of September 2020. MMHg and HgII in ethylated samples are compared against concentrations of MMHg and HgII in blank samples without added Hg.

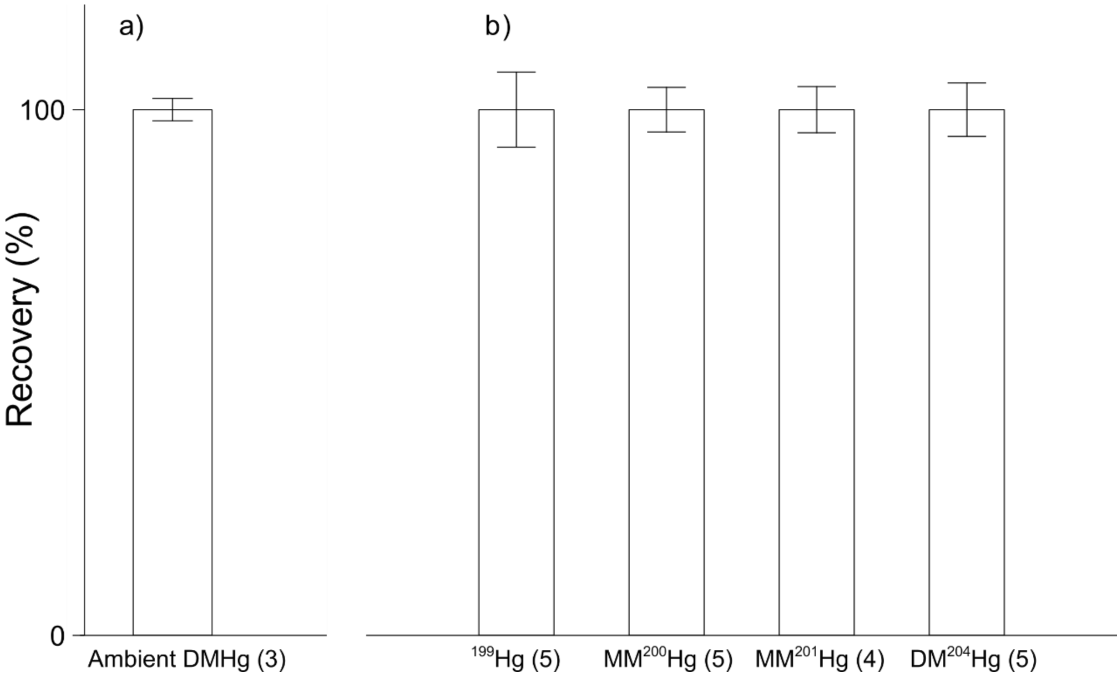

Figure S3. Reproducibility for various Hg species on the Tekran 2700 when added with a) Hamilton syringe b) pipette. Analyzed concentrations ranged between 1.4-2.2 ng L^{-1}.

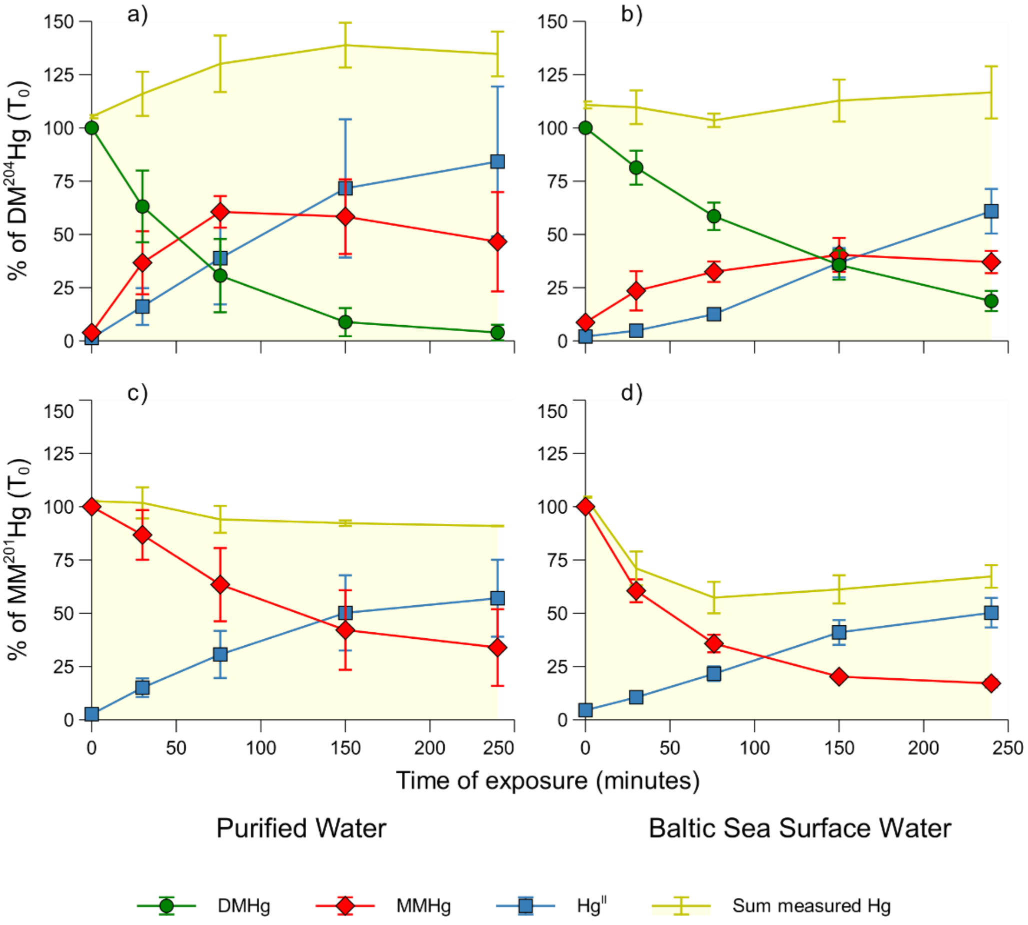

Figure S4. Changes in $DM^{204}Hg$ and $MM^{200}Hg$ concentrations and corresponding photodecomposition products over time for experiment a (SI Table S1). Photodecomposition of $DM^{204}Hg$ in a) purified water and b) in Baltic Sea surface water, and photodecomposition of $MM^{200}Hg$ in c) purified water and d) in Baltic Sea surface water. Sum of measured Hg species includes $DM^{204}Hg$+$MM^{204}Hg$+$^{204}Hg^{II}$ for a) and b) and $MM^{200}Hg$+$^{200}Hg^{II}$ for c) and d). Error bars represent one standard deviation of triplicate incubations.

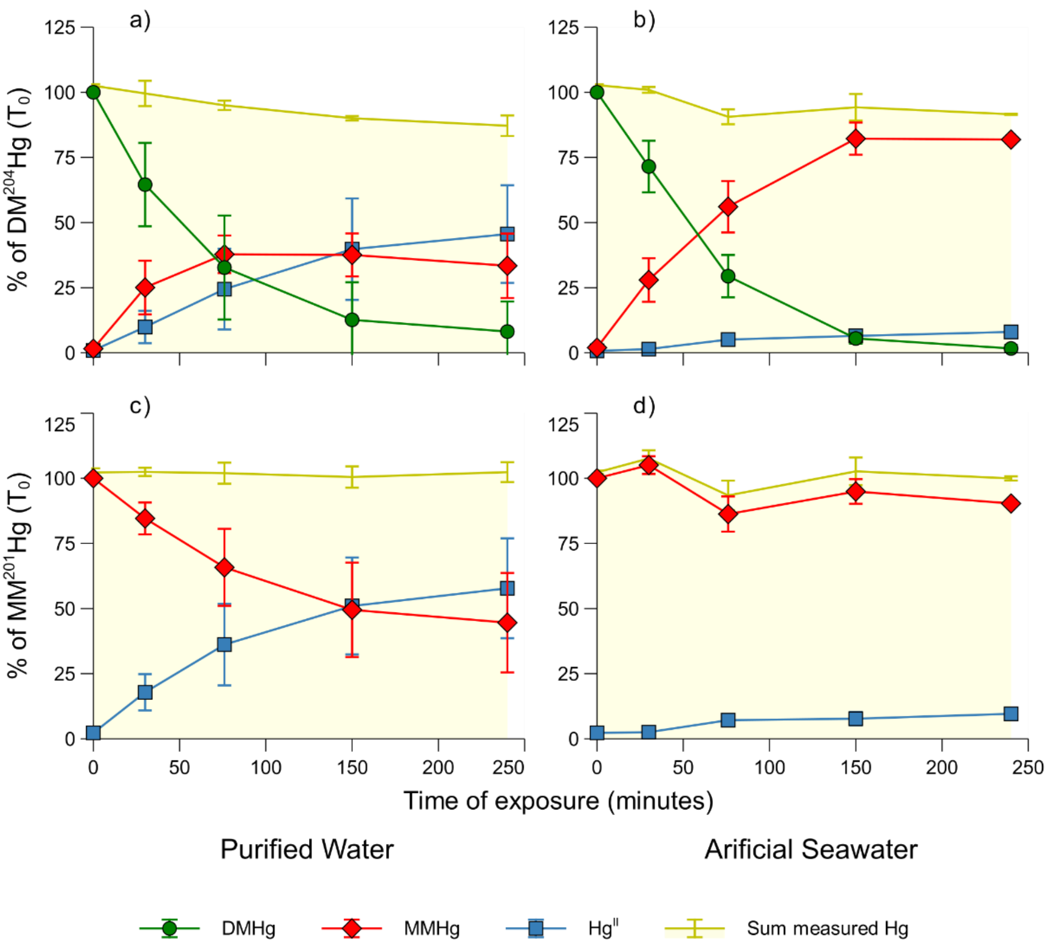

Figure S5. Changes in DM^{204}Hg and MM^{200}Hg concentrations and corresponding photodecomposition products over time for experiment b (SI Table S1). Photodecomposition of DM^{204}Hg in a) purified water and b) in artificial seawater, and photodecomposition of MM^{200}Hg in c) purified water and d) in artificial seawater. Sum of measured Hg species includes DM^{204}Hg+MM^{204}Hg+^{204}Hg^{II} for a) and b) and MM^{200}Hg+^{200}Hg^{II} for c) and d). Error bars represent one standard deviation of triplicate incubations.

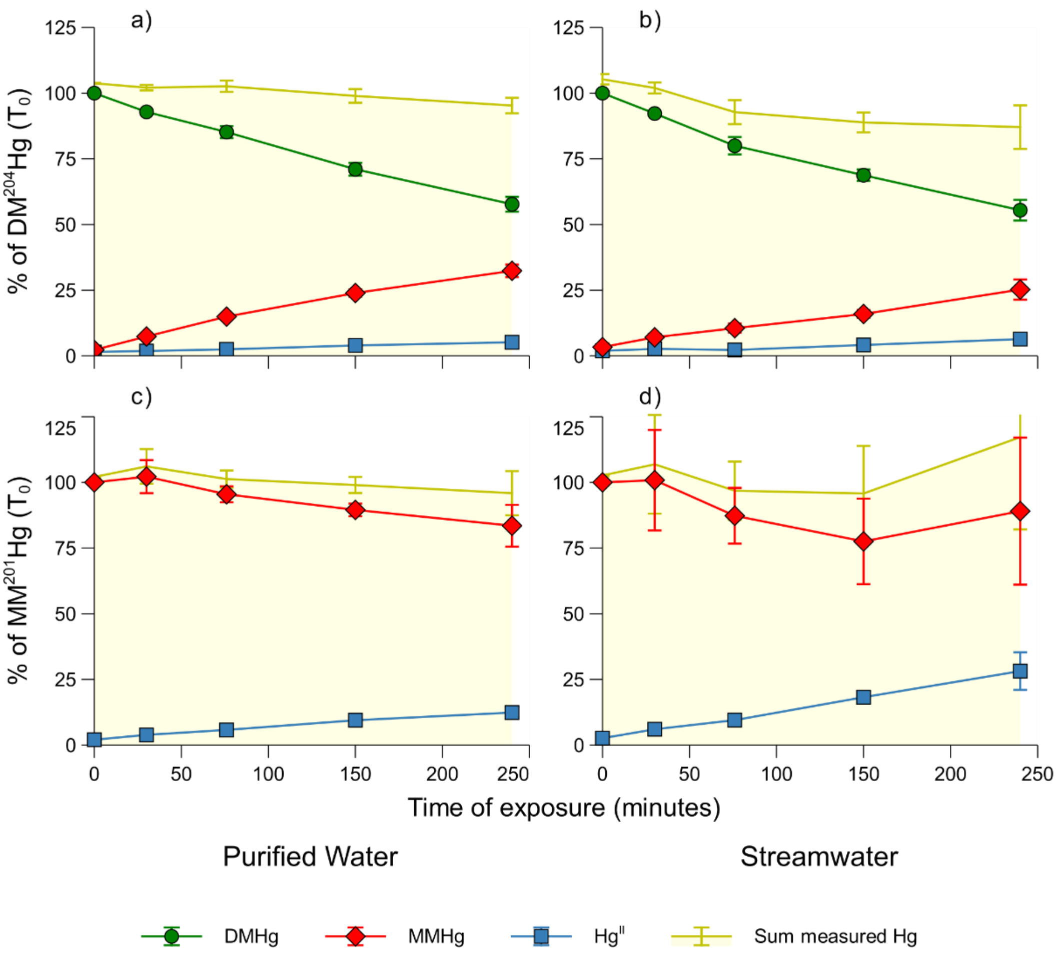

Figure S6. Changes in $DM^{204}Hg$ and $MM^{200}Hg$ concentrations and corresponding photodecomposition products over time for experiment c (SI Table S1). Photodecomposition of $DM^{204}Hg$ in a) purified water and b) in streamwater, and photodecomposition of $MM^{200}Hg$ in c) purified water and d) in streamwater. Sum of measured Hg species includes $DM^{204}Hg+MM^{204}Hg+^{204}Hg^{II}$ for a) and b) and $MM^{200}Hg+^{200}Hg^{II}$ for c) and d). Error bars represent one standard deviation of triplicate incubations.

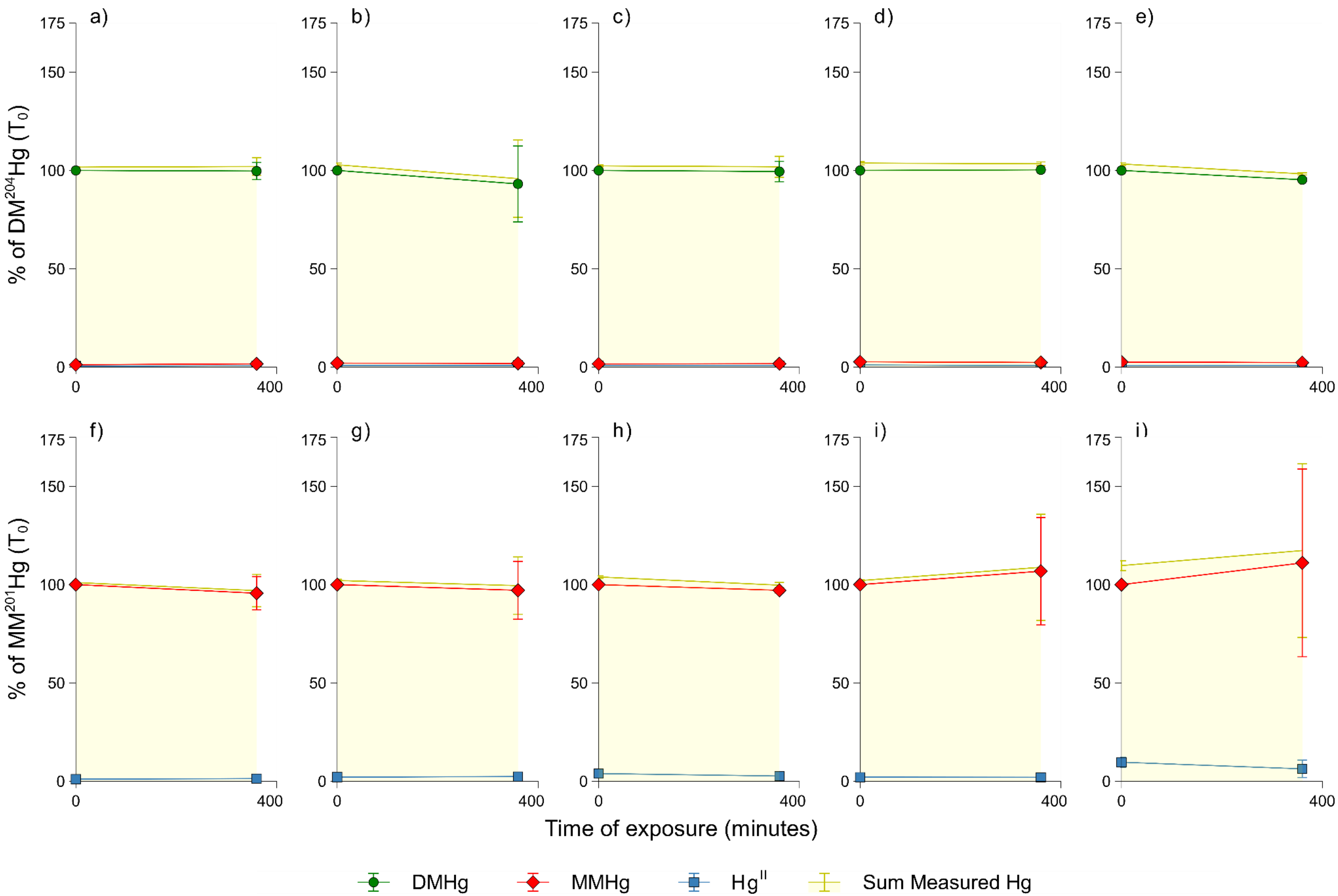
a)
b)
c)
d)
e)
f)
g)
h)
i)
i)
175
150
100
50
0
0
400
% of DM^{204}Hg (T$_0$)
% of MM^{201}Hg (T$_0$)
Time of exposure (minutes)
DMHg
MMHg
Hg^{II}
Sum Measured Hg

Figure S7. Changes in DM^{204}Hg and MM^{200}Hg concentrations and corresponding photodecomposition products over time for dark incubations at 40°C. Panels a-e display concentrations and potential decomposition products for DM^{204}Hg: a) Purified water b) Arctic Ocean surface water c) Baltic Sea surface water d) streamwater e) artificial seawater. Panels f-i display concentrations and potential decomposition products for MM^{200}Hg: a) Purified water b) Arctic Ocean surface water c) Baltic Sea surface water d) streamwater e) artificial seawater. Sum of measured Hg species includes DM^{204}Hg+MM^{204}Hg+^{204}Hg^{II} for a-e and MM^{200}Hg+^{200}Hg^{II} for f-i. Error bars represent one standard deviation of triplicate incubations.

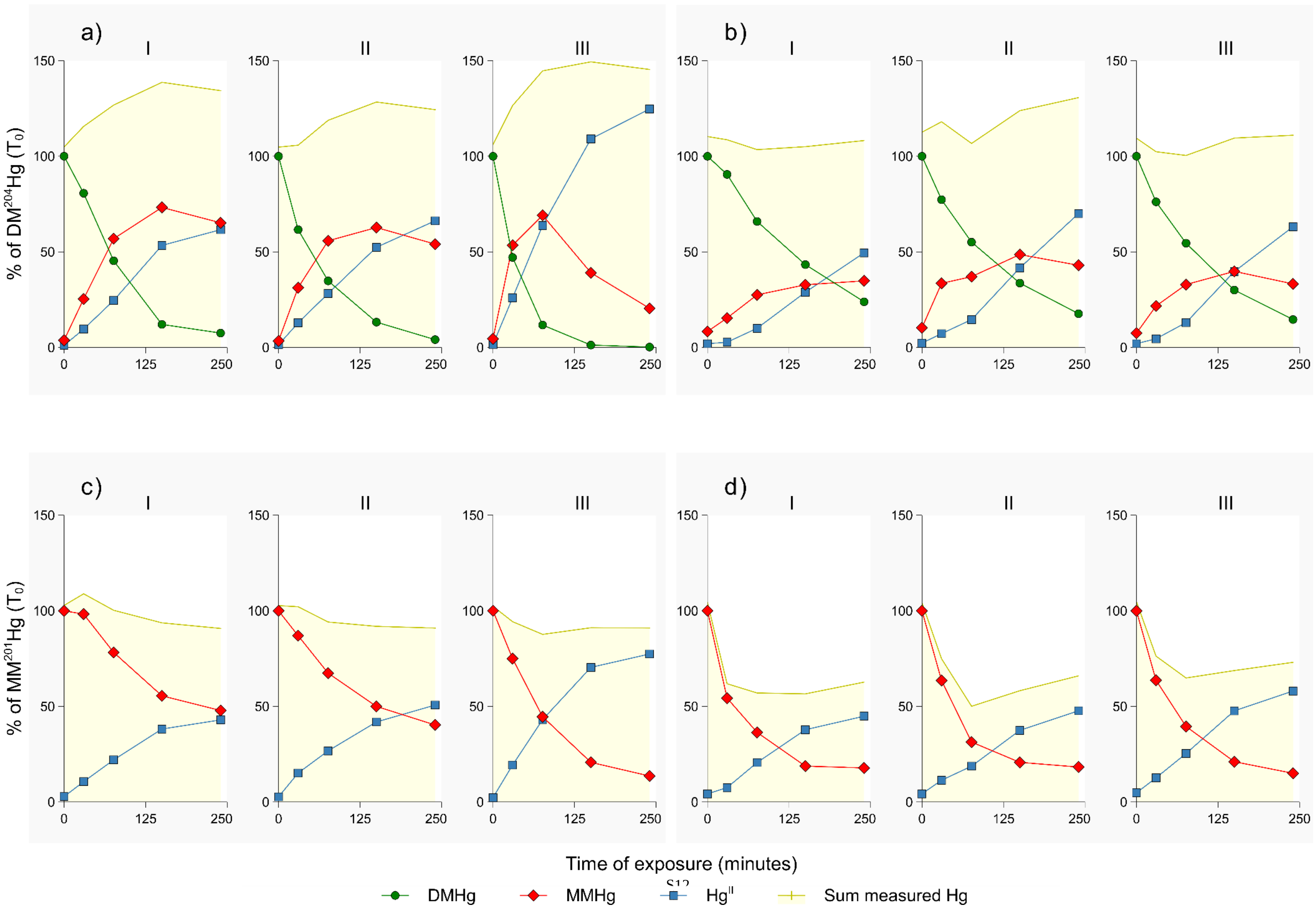
a)
b)
c)
d)
I
II
III
150
100
50
0
125
250
% of DM^{204}Hg (T$_0$)
% of MM^{201}Hg (T$_0$)
Time of exposure (minutes)
DMHg
MMHg
Hg^{II}
Sum measured Hg

Figure S8. Changes in concentrations for the separate incubations from experiment with purified water and Baltic Sea surface water (experiment a, SI Table S1). Roman numbers signify positioning in relation to the lamp. Photodecomposition of $DM^{204}Hg$ in a) purified water and b) in Baltic Sea surface water, and photodecomposition of $MM^{200}Hg$ in c) purified water and d) in Baltic Sea surface water. Sum of measured Hg species includes $DM^{204}Hg+MM^{204}Hg+{}^{204}Hg^{II}$ for a) and b) and $MM^{200}Hg+{}^{200}Hg^{II}$ for c) and d).

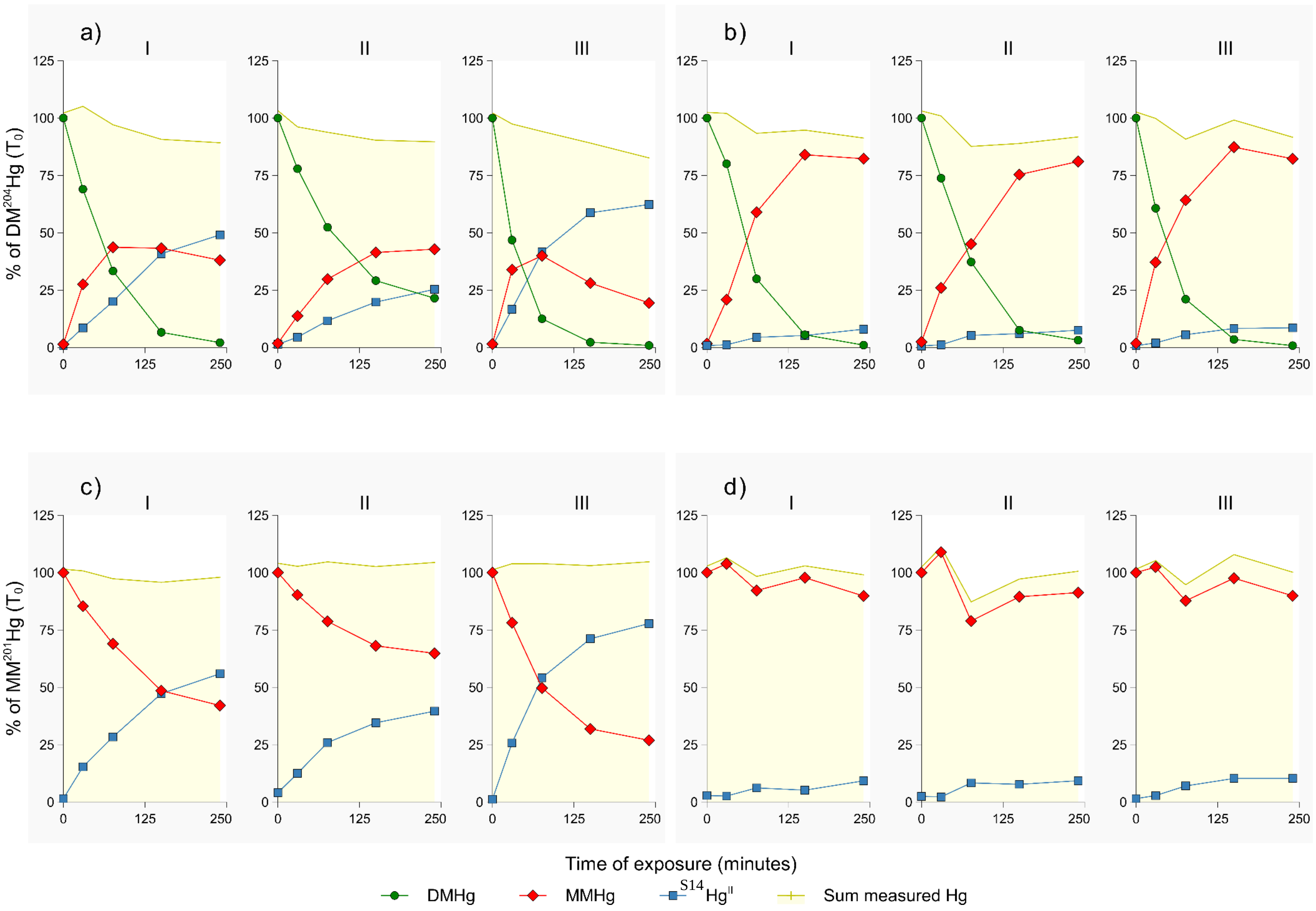
a)
b)
c)
d)
I
II
III
125
100
75
50
25
0
0
125
250
% of DM204Hg (T0)
% of MM201Hg (T0)
Time of exposure (minutes)
DMHg
MMHg
S14HgII
Sum measured Hg

Figure S9. Changes in concentrations for the separate incubations from experiment with purified water and artificial seawater (experiment b, SI Table S1). Roman numbers signify positioning in relation to the lamp. Photodecomposition of $DM^{204}Hg$ in a) purified water and b) in artificial seawater, and photodecomposition of $MM^{200}Hg$ in c) purified water and d) in artificial seawater. Sum of measured Hg species includes $DM^{204}Hg+MM^{204}Hg+{}^{204}Hg^{II}$ for a) and b) and $MM^{200}Hg+{}^{200}Hg^{II}$ for c) and d).

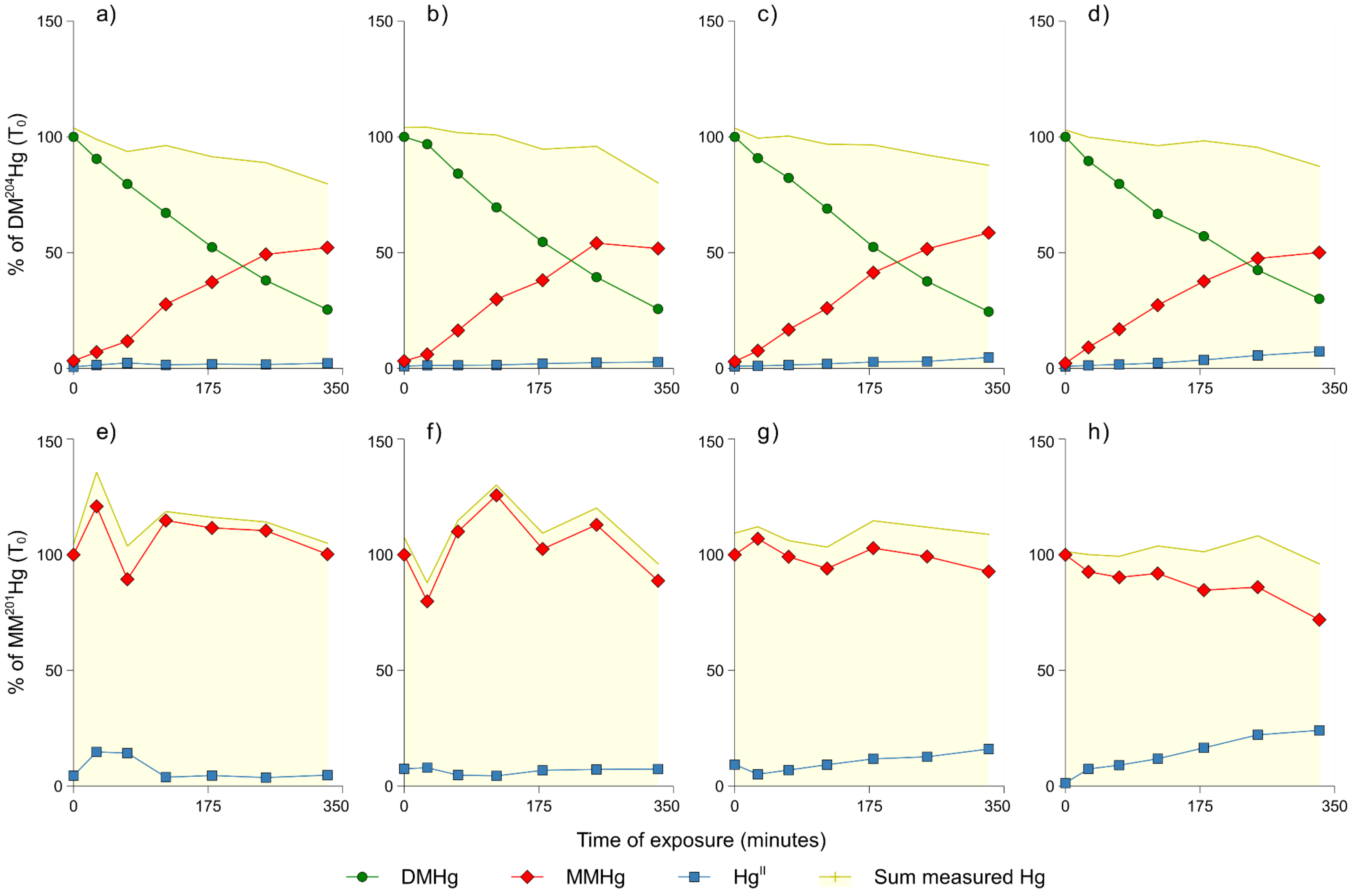
a)
b)
c)
d)
e)
f)
g)
h)
% of DM204Hg (T0)
% of MM201Hg (T0)
0
50
100
150
0
175
350
Time of exposure (minutes)
DMHg
MMHg
HgII
Sum measured Hg

Figure S10. Changes in concentrations in $DM^{204}Hg$ and $MM^{200}Hg$ and corresponding photodecomposition products over time at various DOC concentrations, achieved by mixing artificial seawater with streamwater. Panels a-d display concentrations of and decomposition products for $DM^{204}Hg$ at DOC concentrations of a) 0.9 mg L^{-1} b) 1.0 mg L^{-1} c) 1.3 mg L^{-1} d) 2.4 mg L^{-1}. Panels e-h display concentrations and potential decomposition products for $MM^{200}Hg$ at DOC concentrations of a) 0.9 mg L^{-1} b) 1.0 mg L^{-1} c) 1.3 mg L^{-1} d) 2.4 mg L^{-1}. Sum of measured Hg species includes $DM^{204}Hg$+$MM^{204}Hg$+$^{204}Hg^{II}$ for a) and b) and $MM^{200}Hg$+$^{200}Hg^{II}$ for c) and d). Treatments were prepared without replication.

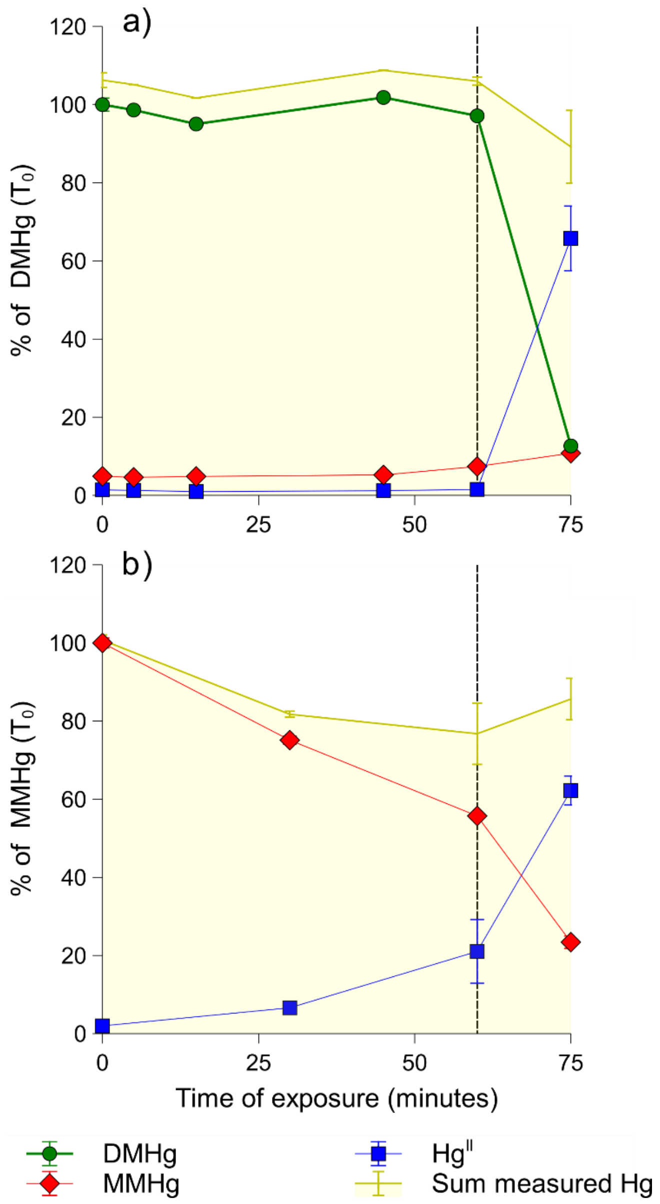

Figure S11. Experiments evaluating the effect of removing the 305nm cutoff filters for photodecomposition of a) DMHg b) MMHg (experiments m+n, Table S1). Black vertical lines represent time point of filter removal. Treatments were prepared without replication. Error bars represent the standard deviation of analysis of replicate subsamples from the same incubation. In a), time points between 5-45 minutes exposure in a) were only subsampled in singlets. In b), all time points were subsampled in duplicates.

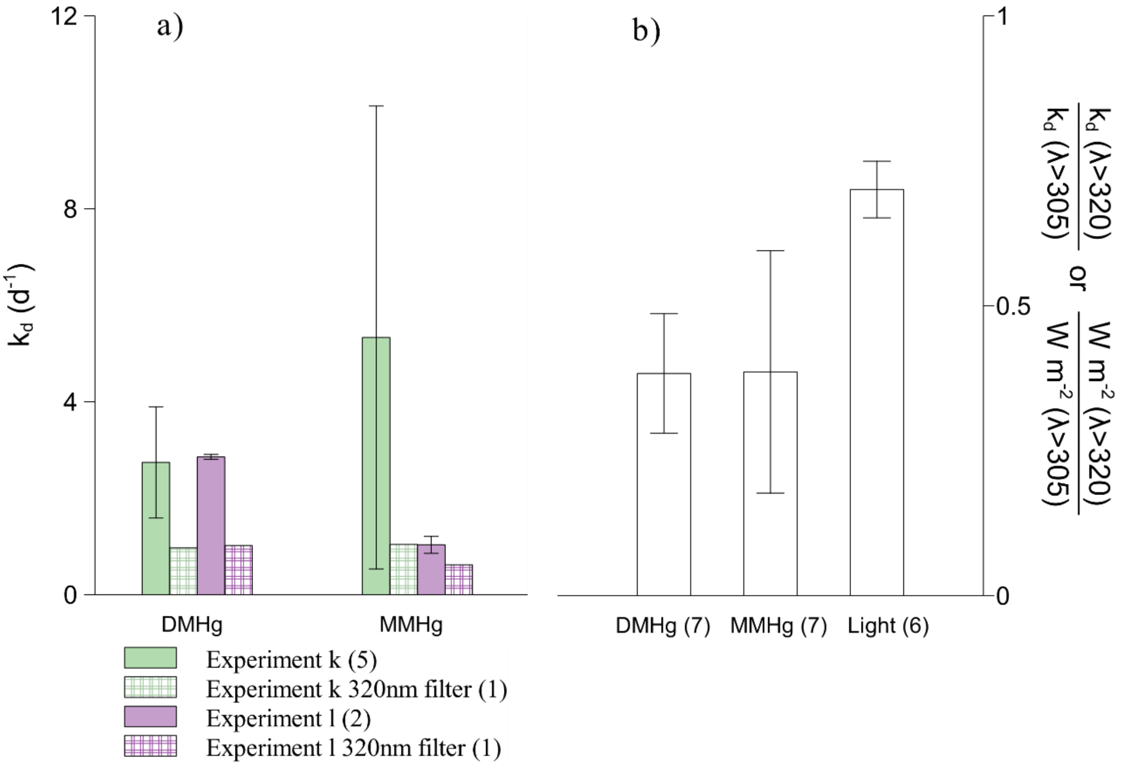

Figure S12. The effect on DMHg and MMHg photodecomposition rates of using 320nm instead of 305nm high-pass filters. a) A comparison of $k_{d\ DMHg}$ and $k_{d\ MMHg}$ values from experiment k (green) and l (purple), when 320 nm (full color) and 305 nm (checkered) cutoff filters were used, respectively. b) Fractions of $k_{d\ DMHg}$ and $k_{d\ MMHg}$ remaining after using 320nm instead of 305nm filters (measured between incubations within the same experiment) compared against the reduction in light intensity (determined using a handheld UV meter from various distances to the lamp). Numbers in parenthesis under the figure specify the number of replicate incubations or UV measuring points. Error bars represent one standard deviation.

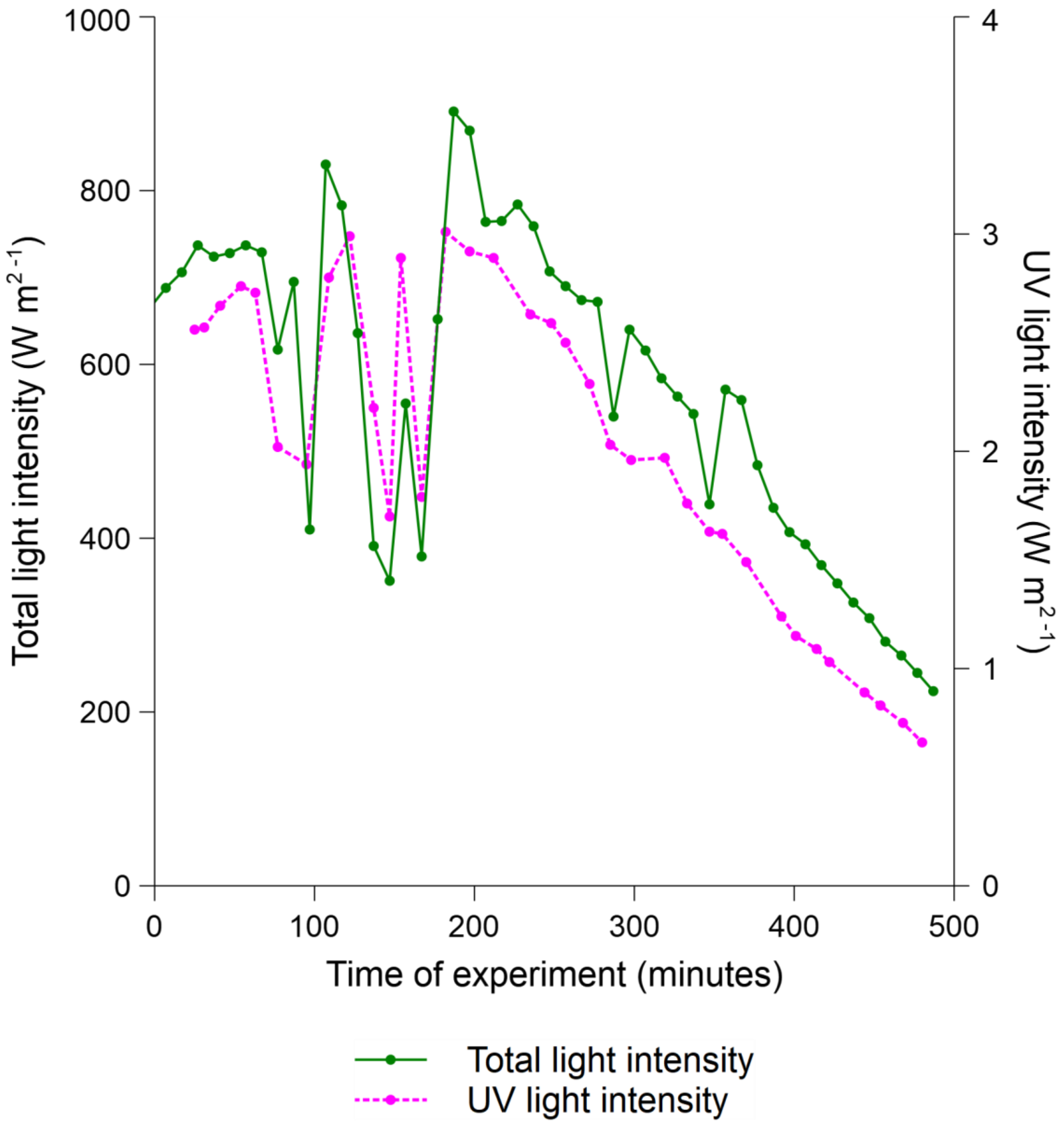

Figure S13. light intensity data captured during the run of experiment f (outdoor incubation). Total light intensity (green solid line) received from MISU weather tower (<100m from experiment location). UV light intensity (pink dashed line) was measured with a hand-held UV meter (LUTRON UV340A). The experiment took place between 10.43-18.43 on a mostly cloud-free day of July.

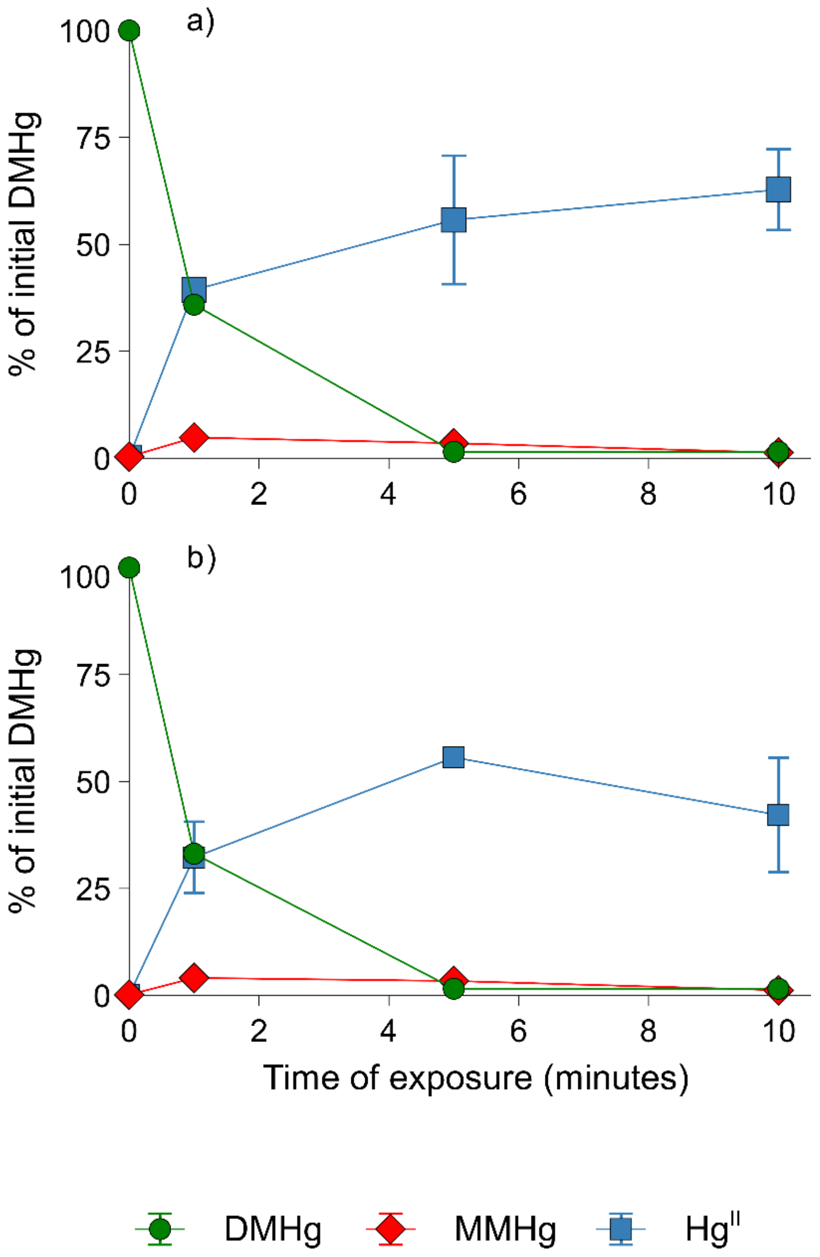

Figure S14. UV lamp exposure incubation with DMHg prepared in THF a) with no buffer b) with 61 µM acetate buffer. This concentration corresponds to ~67 times the concentration added to samples with $DM^{204}Hg$ stock solution. The initial DMHg concentration was ~7 ng L^{-1}. Treatments were prepared without replication. Error bars represent the standard deviation of analysis of triplicate subsamples from the same incubation.

(1) Filippelli, M.; Baldi, F. Alkylation of Ionic Mercury to Methylmercury and Dimethylmercury by Methylcobalamin: Simultaneous Determination by Purge-and-trap GC in Line with FTIR. *Appl. Organomet. Chem.* **1993**, *7* (7), 487–493. https://doi.org/10.1002/aoc.590070707.

(2) Perrot, V.; Jimenez-Moreno, M.; Berail, S.; Epov, V. N.; Monperrus, M.; Amouroux, D. Successive Methylation and Demethylation of Methylated Mercury Species (MeHg and DMeHg) Induce Mass Dependent Fractionation of Mercury Isotopes. *Chem. Geol.* **2013**, *355*, 153–162. https://doi.org/10.1016/j.chemgeo.2013.07.011.

(3) Jiménez-moreno, M.; Perrot, V.; Epov, V. N.; Monperrus, M.; Amouroux, D. Chemical Kinetic Isotope Fractionation of Mercury during Abiotic Methylation of Hg (II) by Methylcobalamin in Aqueous Chloride Media. **2013**, *336*, 26–36. https://doi.org/10.1016/j.chemgeo.2012.08.029.

(4) Perrot, V.; Jimenez-Moreno, M.; Berail, S.; Epov, V. N.; Monperrus, M.; Amouroux, D. Successive Methylation and Demethylation of Methylated Mercury Species (MeHg and DMeHg) Induce Mass Dependent Fractionation of Mercury Isotopes. *Chem. Geol.* **2013**, *355*, 153–162. https://doi.org/10.1016/j.chemgeo.2013.07.011.

(5) Loux, N. T. An Assessment of Thermodynamic Reaction Constants for Simulating Aqueous Environmental Monomethylmercury Speciation. *Chem. Speciat. \& Bioavailab.* **2007**, *19* (4), 183–196.

(6) Karlsson, T.; Skyllberg, U. Bonding of Ppb Levels of Methyl Mercury to Reduced Sulfur Groups in Soil Organic Matter. *Environ. Sci. \& Technol.* **2003**, *37* (21), 4912–4918.

(7) Liem-Nguyen, V.; Skyllberg, U.; Björn, E. Thermodynamic Modeling of the Solubility and Chemical Speciation of Mercury and Methylmercury Driven by Organic Thiols and Micromolar Sulfide Concentrations in Boreal Wetland Soils. *Environ. Sci. \& Technol.* **2017**, *51* (7), 3678–3686.

(8) Skyllberg, U. Competition among Thiols and Inorganic Sulfides and Polysulfides for Hg and MeHg in Wetland Soils and Sediments under Suboxic Conditions: Illumination of Controversies and Implications for MeHg Net Production. *J. Geophys. Res. Biogeosciences* **2008**, *113* (G2).

(9) Baughman, G. L.; Gordon, J. A.; Wolfe, N. L.; Zepp, R. G. *Chemistry of Organomercurials in Aquatic Systems*; US Government Printing Office, 1973.

(10) Diffey, B. L. Sources and Measurement of Ultraviolet Radiation. **2002**, *28*, 4–13.
